# Archaeal histones coordinate conserved DNA sequence motifs to promote CRISPR adaptive immunity

**DOI:** 10.1093/nar/gkag1172

**Published:** 2026-11-10

**Authors:** Pranami Patel, Ariana R Treat, Reyaz Gonzalez, Sucheta Rajaraman, Briana N Cruga, Elizabeth B Chinea, Amarachi Onwuka, Cat Nguyen, Michael P Terns, Andrew Santiago-Frangos

**Affiliations:** Department of Biology, University of Pennsylvania, Philadelphia, PA, 19104, United States; Department of Biology, University of Pennsylvania, Philadelphia, PA, 19104, United States; Department of Biology, University of Pennsylvania, Philadelphia, PA, 19104, United States; Department of Biology, University of Pennsylvania, Philadelphia, PA, 19104, United States; Department of Biology, University of Pennsylvania, Philadelphia, PA, 19104, United States; Department of Biochemistry and Biophysics, University of Pennsylvania, Philadelphia, PA, 19104, United States; Department of Biology, University of Pennsylvania, Philadelphia, PA, 19104, United States; Department of Biochemistry and Biophysics, University of Pennsylvania, Philadelphia, PA, 19104, United States; Department of Biology, University of Pennsylvania, Philadelphia, PA, 19104, United States; University of Georgia, Athens, GA, 30602, United States; Department of Biology, University of Pennsylvania, Philadelphia, PA, 19104, United States

## Abstract

Archaea and bacteria acquire resistance to viruses by integrating fragments of foreign DNA at the beginning of a CRISPR locus. In the archaeon *Pyrococcus furiosus*, histones bind the leader to promote specific integration at the first CRISPR repeat. However, it remains unknown whether archaeal CRISPR leaders contain conserved DNA sequence motifs required for integration. Here, we analyze 542 archaeal CRISPR leaders found across *Thermococcales* and *Methanococcales*. We identify 36 motifs found up to 500 bps upstream of archaeal CRISPR loci. Scrambling or truncating novel motifs or histone binding sites found 100–400 bps upstream of the *P. furiosus* CRISPR modestly decreases integration efficiency. However, mutation of motif T2, which overlaps with the proximal histone site and is adjacent to the CRISPR, ablates both histone-mediated and histone-independent integration. Mutations tiled across T2 reveal that a pyrimidine at position −3 of the leader is required for histone-independent integration, and basepairs −4 to −19 are required for histone-mediated integration. T2 is found in most *Thermococcales*, and a similar sequence is found in most *Methanococcales* leaders, suggesting this motif may be a broadly conserved element that facilitates CRISPR adaptive immunity across taxonomic orders. These data shed light on the sequence requirements for histone-mediated DNA integration

## Graphical Abstract

**Graphical Abstract:**
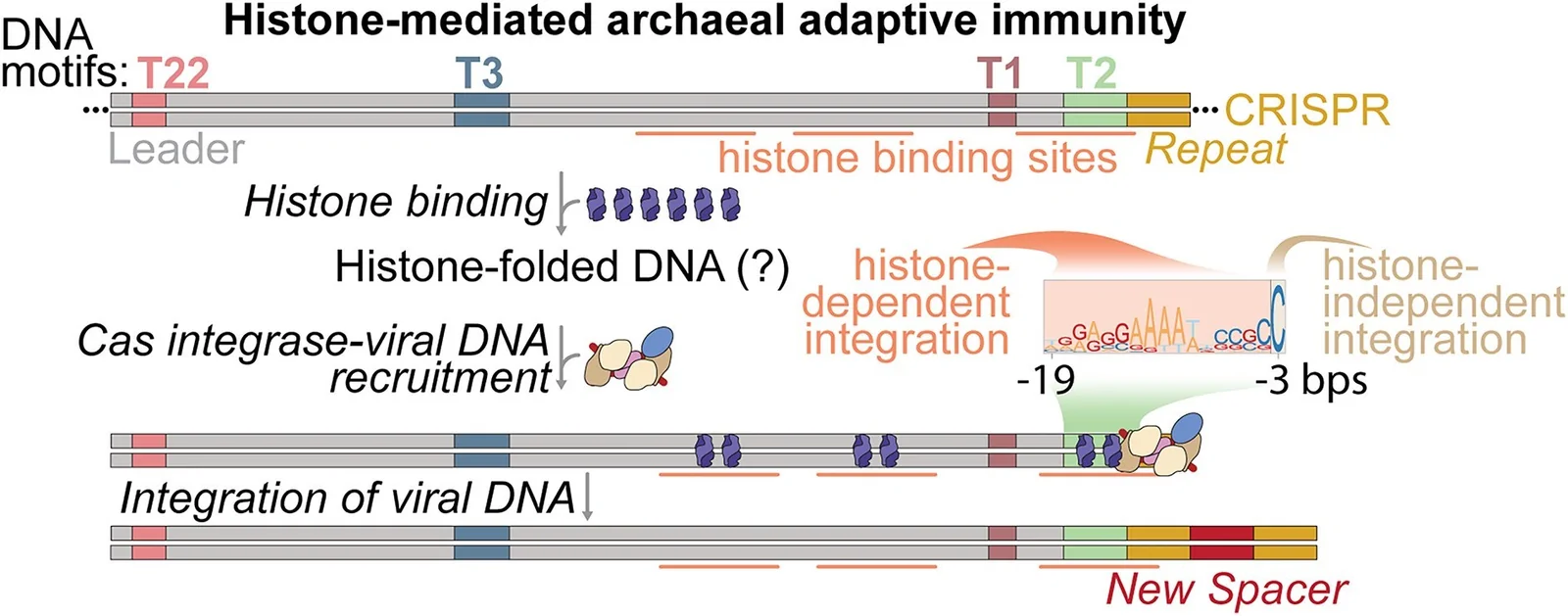

## Introduction

Archaea and bacteria use the CRISPR adaptive immune system to defend themselves from phage infection [1]. To acquire immunity, the CRISPR-associated integrase (Cas1-2) captures fragments of viral DNA (“prespacers”) and integrates these at a repeated DNA sequence in the CRISPR locus, called the “repeat” [2, 3]. Many CRISPR systems encode a nuclease (Cas4) that recognizes and cleaves a Protospacer Adjacent Motif (PAM) within the prespacer that is needed for subsequent targeting of viral DNA during interference and assists in integrating the prespacer in the correct orientation [4–8]. CRISPR loci can accumulate tens to hundreds of spacers and repeats [9]. However, most Cas integrases preferentially integrate new viral DNA prespacers at the first repeat within a CRISPR locus, ensuring efficient transcription and processing of CRISPR RNAs that target the most recently encountered phage [10, 11]. In bacteria from the phylum *Pseudomonadota*, the nucleoid-associated protein Integration Host Factor (IHF) binds and folds DNA upstream of the CRISPR (“leader”) into a distinct shape, consisting of a U-turn often followed by a loop, which recruits the Cas integrase to the first CRISPR repeat specifically [12–15]. However, it remains unclear how bacterial and archaeal phyla lacking IHF may use other DNA bending proteins to fold the leader DNA into shapes that promote CRISPR adaptive immunity.

Many archaea encode histones that package their genome [16–20]. Archaeal histones can uniquely form “hypernucleosomes” that consist of multiple repeating units of a 30 bp-bound histone dimer, held together by protein-protein interactions between dimers both across and along the axis of the DNA–protein superhelix [16, 20, 21]. A recent study has shown that the archaeon *Pyrococcus furiosus* uses histones to directly promote CRISPR integration at the first repeat (Fig. 1A) [22]. *P. furiosus* encodes two histone subunits, Histone A and B, which can each increase the number of new spacers integrated at the first CRISPR repeat independently [22]. Although deletion of Histone A has a greater impact on *in vivo* integration efficiency than deletion of Histone B [22]. *P. furiosus* histone tetramers bind to three roughly 60 basepair (bp) regions within the leader. These binding sites range from partially overlapping with the first repeat by 10 bps, to up to 234 bps upstream of the first repeat (Fig. 1A). The first 250 bps of the leader are strongly conserved across the seven CRISPR loci found in *P. furiosus* DSM 3638, and these three histone binding sites are frequently occupied and protected from nuclease digestion in all leaders [22]. Additionally, a previous bioinformatic analysis identified several conserved motifs within the first 200 bps of archaeal type I-B CRISPR leaders [23], some of which may play a role in integration. However, other computational analyses have suggested that archaeal leaders may be up to 500 bps long [9, 24]. Indeed, up to 400 bps of the type I-A archaeal leader from *Saccharolobus solfataricus* has been shown to promote efficient integration of new spacers at the first CRISPR repeat [4]. Therefore, it remains unclear what regions of the leader regulate CRISPR adaptation in *P. furiosus* and whether Cas4, which is likely associated with Cas1-2 during the first of two successive transesterification reactions, assists in recognition of the leader [25].

**Figure 1.**
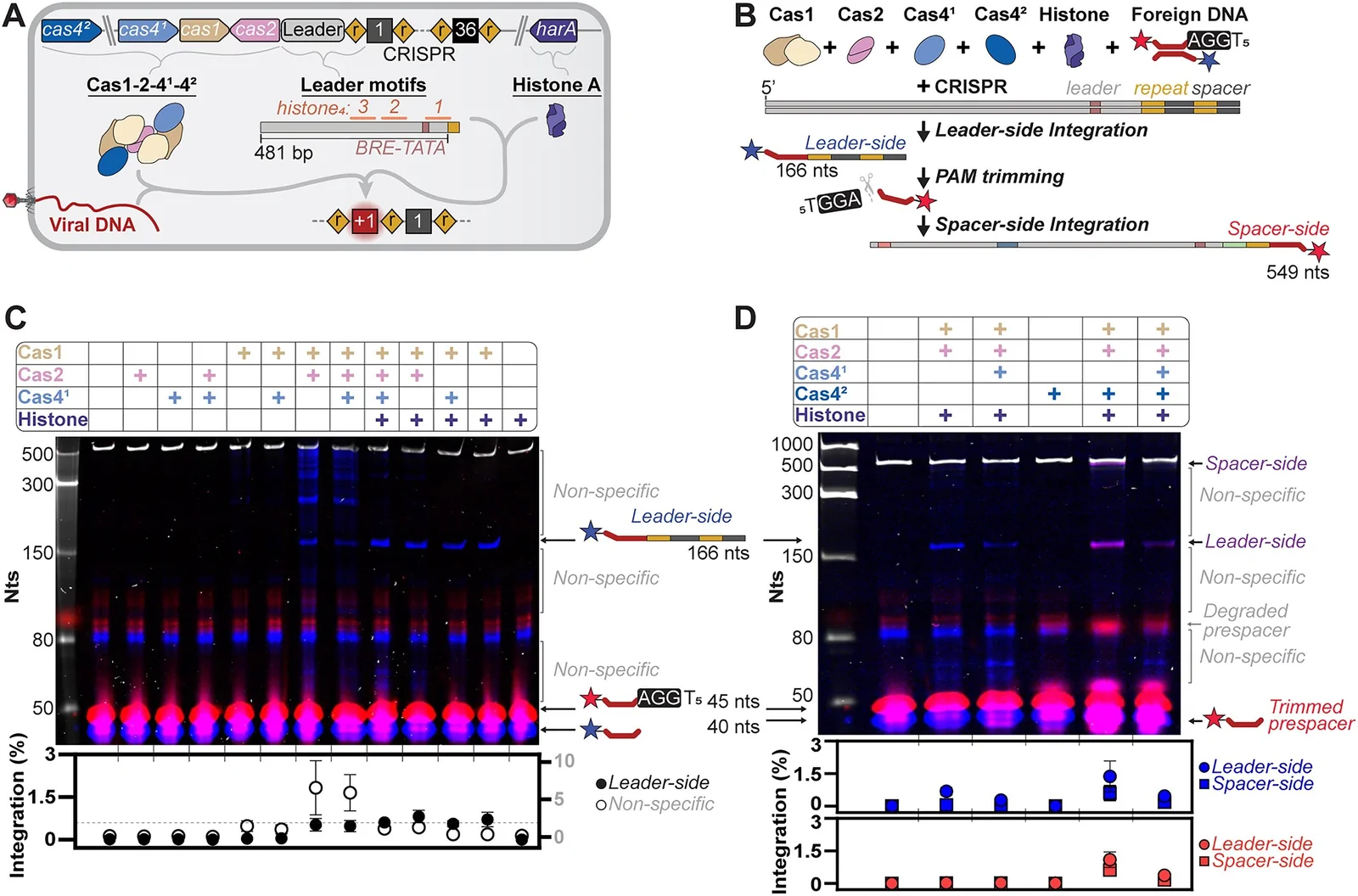
Reconstitution of histone-mediated CRISPR adaptation *in vitro*.(**A**) Scheme of the CRISPR adaptation machinery from the archaeon *P. furiosus* and the minimal components known to be required for the Cas-dependent integration of new spacers into the CRISPR locus. Leader motifs with known functions, such as three histone tetramer binding sites (orange), and a binding site for transcription factor II B and TATA box binding protein (overlapping the first histone binding site) are depicted. (**B**) Scheme depicting the substrates used to reconstitute CRISPR adaptation in a test tube, and the expected integration products. The psPAM-5T prespacer consists of a strand containing the “AGG” PAM with a 5 nt-long thymidine overhang, and a non-PAM strand that has been trimmed. Each strand is labeled with different fluorophores for strand-specific tracking of integration events. (**C**) Integration reactions in which each protein, excluding Cas42, is added in a combinatorial manner to reveal the requirements for site-specific integration. Reactions were resolved on a denaturing urea–PAGE gel. The ladder and unlabeled CRISPR fragment substrate were visualized with SYBR Safe (white), the prespacer non-PAM strand is labeled with IRDye^®^ 700 (blue), and the PAM-containing strand is labeled with IRDye^®^ 800 (red). A representative of three replicate gels is shown (Supplementary Fig. S4). The quantification of three independent replicate reactions is shown per lane in the plot below the gel. Mean and standard deviations are shown. To facilitate comparison, a horizontal dashed line is drawn across the integration plot at a *y*-axis value corresponding to integration efficiency for a reaction containing all components required for specific leader-side integration. The percent of leader-side integration is plotted relative to the left *y*-axis, and non-specific integration is plotted on the right *y*-axis. A roughly 80 nt-long labeled band appears in most lanes in a protein-independent manner, and may be a contaminant in the original synthesized DNA (IDT). (**D**) Integration reactions in which each protein, including Cas42, is added in a combinatorial manner to reveal the requirements for site-specific integration. A representative of three replicate gels is shown (Supplementary Fig. S6). The quantification of three independent replicate reactions is shown per lane in the plot below the gel. Mean and standard deviations are shown. Leader- (circles) and spacer-side (squares) integration events have been subdivided into those corresponding to integration of the non-PAM (blue) and PAM-containing (red) strands. Cas42 is a promiscuous and highly active DNase which trims long prespacers to an integrase protected size (roughly 37 nts), and degrades the fluorescently labeled prespacers to generate products that migrate faster and slower through the polyacrylamide gel (Supplementary Fig. S5).

Here, we analyzed 542 non-redundant CRISPR loci from the archaeal taxonomic orders *Thermococcales* and *Methanococcales*. This analysis identified 36 conserved DNA sequence motifs, most of which are specific to either *Thermococcales* (T1-T22) or *Methanococcales* (M1-M14) CRISPR leaders. *Thermococcales* leaders are generally longer, containing conserved motifs up to 500 bps upstream of the CRISPR (Fig. 2). Whereas *Methanococcales* leaders tend to be shorter, <350 bps in length. To determine the role of leader motifs in integration, we reconstituted *P. furiosus* CRISPR adaptation *in vitro*. Integration reactions with successively truncated leaders reveal that up to 403 bps of the *P. furiosus* leader is required for efficient and specific integration at the first repeat. Scrambling of most upstream motifs, or of the distal and middle histone binding sites, results in modest decreases in integration efficiency. However, scrambling a 30 bp motif, T2, that is adjacent to the repeat and which overlaps with the proximal histone binding site ablates both histone-mediated and histone-independent integration at the first CRISPR repeat. Additional 3–4 bp tiled mutations reveal that the 3rd bp of the leader (“-3”) is critical for histone-independent integration and is likely contacted directly by Cas1 during integration, as has been seen in the type I-F Cas1 integrase [15]. Further, a later segment of T2, bps −4 to −19 of the leader, is required for histone-dependent integration. The T2 motif, which is essential for integration, is conserved through most *Thermococcales* CRISPRs, and a similar motif is found at the same position in most *Methanococcales* leaders. Collectively, these data show that *P. furiosus* histones bind the leader and coordinate up to 403 bps of upstream DNA to promote site-specific integration of viral DNA at the first CRISPR repeat.

## Materials and methods

### Nucleic acid preparation

The fluorescent splayed foreign DNA mimics, “prespacers,” used in integration assays (Fig. 1 and Supplementary Table S1) were synthesized and resuspended in 1x TE buffer (10 mM Tris-HCl pH 8, 1 mM EDTA) before use. The “sense” strand containing the 3′ PAM prior to Cas4-mediated processing is labeled with 5′ IRDye^®^ 700, and the “anti-sense” strand is labeled with 5′ IRDye^®^ 800 (IDT). To make CRISPR integration substrates, the sequences consisting of the CRISPR leader, the first two CRISPR repeats and two spacers were first synthesized and cloned into pUC57 (Gene Universal). Plasmids containing the wild-type and mutant CRISPRs have been made available on Addgene (Supplementary Table S2). Primer pairs were designed to amplify fragments of DNA containing the first two CRISPR repeats, the first spacer and approximately half of the second spacer, and different amounts of the leader (Supplementary Table S1), using Q5 DNA polymerase (NEB). After PCR, the template plasmid was removed by restriction enzyme digest with DpnI (NEB) at 37°C, and the PCR amplification products were purified using the DNA Clean and Concentrator Kit (Zymo Research).

### Expression and purification of pyrococcus furiosus integration complex proteins

The *P. furiosus* Cas1, Cas2, Cas4^1^, Cas4^2^, and Histone A genes were codon-optimized for *Escherichia coli* expression, synthesized and cloned into expression vectors (Supplementary Table S2). Cas2 and Histone A were expressed without a tag, whereas Cas1 was expressed with an N-terminal translational ramp, a 10x-His, and SUMO tag. Cas4^1^ and Cas4^2^ were expressed with an N-terminal 6x-His and SUMO tag. The final constructs were verified by Sanger sequencing. Cas1, Cas2, and Histone A expression plasmids were individually transformed into *E. coli* One Shot BL21-AI, chemically competent cells (Invitrogen). Cultures were grown in LB-Miller media (10 g/l Tryptone, 10 g/l NaCl, and 5 g/l yeast extract) supplemented with the corresponding antibiotics (Supplementary Table S2) and incubated at 37°C while shaking at 250 rpm (rotations per minute) until the OD_600_ reached 0.6, after which they were cooled for 1 h at 4°C. Protein expression was then induced with the addition of 0.2% (w/v) L-arabinose and 0.5 mM IPTG (isopropyl-b-d-thiogalactoside). The cultures were then incubated at 25°C with shaking at 225 rpm overnight, and cells were harvested by centrifugation at 5000 rpm for 10 min at 4°C. Cas4^1^ and Cas4^2^ overexpression plasmids were each individually co-transformed with plasmid pDB1282, which overexpresses proteins involved in the biosynthesis and incorporation of iron–sulfur clusters into target proteins [26–28], into *E. coli* BL21-AI cells (Invitrogen). Cas4^1^ and Cas4^2^ cultures were grown in 2-YT Broth media, supplemented with antibiotics, and incubated at 37°C with shaking at 250 rpm (rotations per minute) until the OD_600_ reached 0.3–0.5. Then, the media was supplemented with final concentrations of 25–50 µM FeCl_3_, 300 µM L-cysteine, and 0.2% (w/v) L-arabinose, and then further incubated at 37°C and 250 rpm. When OD_600_ reached 0.6–0.8, the culture was cooled for 1 h at 4°C, then supplemented with 0.1 mM IPTG. To reduce headspace, respective cultures were combined to 2 l of culture per 2 l flask. Protein expression then occurred at 16°C with shaking at 180 rpm overnight, and cells were harvested as above.

Cas1 was purified by first resuspending the cell pellets in lysis buffer (50 mM HEPES pH 7.5, 500 mM KCl, 10 mM imidazole, and 5% glycerol), at a ratio of 5 ml buffer per 1 g of pellet, supplemented with 0.3× Halt Protease Inhibitor Cocktail (Thermo Fisher). Cells were lysed via sonication and clarified by centrifugation at 40 000 *g*, for 20 min, at 4°C. Clarified lysate was then flowed over HisTrap HP resin (Cytiva) to affinity purify His-tagged Cas1. The HisTrap HP resin was washed with 20 column volumes of lysis buffer. Tagged Cas1 was then eluted using lysis buffer containing 500 mM imidazole. Cas1 was concentrated at 4°C (Corning Spin-X concentrators). The N-terminal ramp, 10x-His, and SUMO tag were cleaved from Cas1 with in-house Sentrin-specific protease 2 (SENP2), overnight at 4°C while dialyzing into a buffer containing 20 mM HEPES pH 7.5, 250 mM monopotassium glutamate, and 2% glycerol. Cleaved Cas1 was partially separated from contaminants using a HisTrap HP column equilibrated in 50 mM HEPES pH 7.5, 250 mM monopotassium glutamate, 20 mM imidazole, and 5% glycerol, and eluted with a gradient into an otherwise identical buffer containing 500 mM imidazole. Fractions containing cleaved Cas1 were further purified by size exclusion chromatography (SEC) on a HiLoad Superose 6 16/600 column (Cytiva) equilibrated in 20 mM HEPES pH 7.5, 250 mM monopotassium glutamate, and 2% glycerol. Cas1-containing fractions were concentrated, aliquoted, and stored at −80°C.

Cas2 was purified by first resuspending the cell pellets in lysis buffer (80 mM HEPES pH 7.5, 200 mM monopotassium glutamate, and 5% glycerol) at a ratio of 5 ml buffer per 1 g of pellet. Cells were lysed via sonication and clarified by centrifugation at 45 000 *g* for 20 minutes at 4°C. The lysate was then heat-denatured at 85°C for 20 min, and then centrifuged again at 45 000 *g* for 20 min at 4°C. Ammonium sulfate salt was slowly dissolved into the clarified lysate to a final saturation of 45%, equilibrated for 1 h at 4°C, and then clarified by centrifugation at 20 000 *g* for 10 min at 4°C. Additional ammonium sulfate was then added to the clarified lysate to further increase the saturation to 60%, and the lysate was then re-centrifuged. The pellet from this centrifugation was then resuspended in buffer containing 50 mM HEPES pH 7.5 and 5% glycerol, at a ratio of 75 ml buffer per 1 gram of protein pellet. The sample was then loaded onto a HiTrap Capto Q column (Cytiva) that was equilibrated and washed with a low-salt buffer of 50 mM HEPES pH 7.5, 100 mM monopotassium glutamate, and 5% glycerol. Cas2 flowed through the HiTrap Capto Q column and was loaded onto a HiTrap SP HP column (Cytiva), which it also flowed through. Remaining contaminants were further separated from untagged Cas2 by a final purification by SEC on a HiLoad Superose 6 16/600 column (Cytiva) equilibrated in 20 mM HEPES (pH 7.5), 200 mM monopotassium glutamate, and 2% glycerol. Cas2- containing fractions were concentrated, aliquoted, and stored at −80°C.

Histone A was purified by first resuspending the cell pellets in lysis buffer (50 mM HEPES pH 7.5, 100 mM monopotassium glutamate, and 5% glycerol) at a ratio of 5 ml buffer per 1 g of pellet. Cells were lysed via sonication and clarified by centrifugation at 45 000 *g* for 20 min at 4°C. MgCl_2_ and CaCl_2_ were added to final concentrations of 5 and 1.25 mM, respectively, to the clarified lysate, which was then treated with 0.5 µl of Turbo DNase I (Thermo Fisher) per 1 ml of lysate at 37°C for 1 h. The lysate was then heat-denatured at 70°C for 30 min and clarified by centrifugation at 45 000 *g* for 20 min at 4°C. The supernatant was loaded onto a HiTrap Heparin HP column equilibrated in a buffer containing 50 mM HEPES pH 7.5, 100 mM monopotassium glutamate, and 5% glycerol and eluted via a linear gradient to a buffer containing 50 mM HEPES (pH 7.5), 2 M KCl, and 5% glycerol. Histone A-containing fractions were further purified by SEC on a HiLoad Superose 6 16/600 column (Cytiva) equilibrated in 20 mM HEPES (pH 7.5), 200 mM KCl, and 2% glycerol. Histone A-containing fractions were concentrated, aliquoted, and stored at −80°C.

For Cas4^1^ and Cas4^2^ purification, the cells were resuspended in lysis buffer (50 mM HEPES pH 7.5, 500 mM KCl, 10% glycerol, and 20 mM imidazole) that was first autoclaved to reduce dissolved oxygen, before supplementing with 5 mM TCEP, 0.3x Halt Protease Inhibitor Cocktail (Thermo Fisher), 0.1% Triton-X, 200 uM FeCl_3_, 1 mM L-cysteine, and 1 mM pyridoxal phosphate. Cells were lysed via sonication and clarified by centrifugation at 45 000 *g*, for 45 min, at 4°C. Clarified lysate was transferred to an anaerobic Coy chamber (95%–97% N₂, 3%–5% H₂ atmosphere), for all subsequent purification steps. All buffers and solvents used throughout the purification were autoclaved and then degassed in the chamber for three days prior to use. Lysate was loaded on a HisTrap HP column (Cytiva) in lysis buffer and eluted with the same buffer containing 500 mM imidazole. The eluate was collated, and then the N-terminal tag was cleaved from either Cas4 protein by digestion with SENP2 overnight at 25°C, while dialyzing in the anaerobic chamber into a buffer containing 50 mM HEPES pH 7.5, 500 mM KCl, 10% glycerol, and 5 mM TCEP. The cleaved Cas4 preparations were then filtered through a 0.22 µM syringe filter unit, prior to loading onto a HisTrap HP column (Cytiva) equilibrated with lysis buffer. The HisTrap HP column was washed with lysis buffer. For Cas4^1^, lysis buffer containing up to 95 mM imidazole was used to wash the column, and Cas4^1^ was eluted with lysis buffer containing 120–500 mM imidazole. For Cas4^2^, the cleaved and untagged Cas4^2^ protein flowed through the HisTrap HP column, and additional untagged Cas4^2^ protein was removed from the column by washing with lysis buffer containing up to 95 mM imidazole. Cas4^1^- and Cas4^2^-containing fractions were separately collated and concentrated, removed from the anaerobic chamber, and further purified by SEC on a Superdex 75 10/300 GL column (Cytiva) that had been equilibrated with degassed Cas4 storage buffer [50 mM HEPES (pH 7.5), 500 mM KCl, 10% glycerol, and 5 mM DTT (dithiothreitol)]. Cas4-containing SEC fractions were immediately collated and concentrated as they eluted from the SEC column, aliquoted, and snap-frozen in liquid nitrogen prior to storage at −80°C.

An alternative method to purify Cas4^1^ was designed to further limit exposure to aerobic environments, which generated a protein stock that was used for experiments shown in Fig. 3. This protein stock was slightly less pure but had slightly higher iron content (Supplementary Fig. S1). However, it still appeared inactive and did not trim the PAM from the prespacer under the conditions used here. This alternative Cas4^1^ purification starts with resuspension of the cell pellet with the following lysis buffer: 50 mM HEPES pH 7.5, 500 mM KCl, 5% glycerol, and 20 mM imidazole. This buffer was first autoclaved to reduce dissolved oxygen, before supplementing with 10 mM TCEP. Affinity purification was performed as described above for both Cas4 proteins. The eluate from the HisTrap HP column was diluted 10-fold into a low salt version of the lysis buffer and was loaded onto a HiTrap Heparin HP column (Cytiva) equilibrated with a buffer containing: 20 mM HEPES pH 7.5, 50 mM KCl, 2% glycerol, and 10 mM TCEP. Cas4^1^ was eluted in a linear gradient to a storage buffer containing 20 mM HEPES pH 7.5, 500 mM KCl, 2% glycerol, and 10 mM TCEP, and this alternative Cas4^1^ preparation was subsequently aliquoted and stored at −80°C.

### Quantification of iron content of purified Cas41 and Cas42

Purified Cas4^1^ and Cas4^2^ have a brown color characteristic of iron–sulfur cluster-containing proteins. To estimate the amount of iron-bound Cas4^1^ and Cas4^2^, the iron was quantified using the Total Iron-Binding Capacity and Serum Iron Assay Kit (Cayman Chemical, item # 702870). The supplied protocol was adapted into the following method. Briefly, a FeCl_3_ dilution series was made ranging from 2.5 to 100 µM, by dilution in a buffer that recapitulates the Cas4 protein stock storage buffer. Prior to measurement with the manufacturer’s Total Iron-Binding Capacity and Serum Iron Assay Kit, aliquots of purified Cas4 stocks were subjected to acid hydrolysis to release protein-bound iron [29]. FeCl_3_ dilution standards were treated in the same manner. 50 µl of each standard or protein dilution were mixed with 10 µl of 6 M HCl and incubated at 80°C for 10 min. Acid-treated samples were then cooled to 22°C for 5 min and centrifuged at 500 *g* for 2 min to collect condensate. If a black precipitate was observed, samples were centrifuged for an additional 6 min and only the clear supernatant was used for analysis. The acid-treated samples were then neutralized through the addition of 10 µl of 6 M NaOH. Following neutralization, standards and samples were used as input for the Total Iron-Binding Capacity and Serum Iron Assay Kit per the manufacturer’s instructions. 50 µl of each FeCl_3_ standard dilution, 50 µl of Serum Iron Buffer (blank), or 50 µl of diluted Cas4, was mixed with 150 µl of Serum Iron buffer in a 96-well plate. Samples were incubated and shaken in the BioTek Epoch 2 Microplate Spectrophotometer for 9 min and 30 s at 37°C. Absorbance at 570 nm was measured for each sample (“Abs1”). Then, 50 µl of Iron Color Reagent was added to each sample, and reactions were mixed and incubated in the spectrophotometer in the dark at 37°C for 30 min. Then, the absorbance at 570 nm was measured for each sample (“Abs2”). The change in absorbance was calculated, (*Δ Abs = Abs2 - Abs1*), and averaged for duplicate standards and samples. The corrected average absorbance for the FeCl_3_ standards was calculated by subtracting the readings for Serum Iron Buffer alone, whereas the corrected absorbance for Cas4-containing samples was calculated by subtracting the readings for Cas4 storage buffer. These corrected absorbance values were used to generate a standard curve, and a linear fit to these values was used to calculate the concentration of iron in the Cas4^1^ and Cas4^2^ samples (Supplementary Fig. S1).

### *In vitro* integration assays

Integration assays were performed in triplicate. Two initial reaction tubes were prepared separately. The first tube contained 1000 nM Cas1, 1000 nM Cas2, 250 nM Cas4^1^, and 50 nM of fluorescently labeled prespacer (Supplementary Table S1). When appropriate, the first tube also contained 50 nM Cas4^2^. The second tube contained 50 nM of a PCR fragment consisting of the leader and first two repeats of the CRISPR locus encoded adjacent to Cas2 in the *P. furiosus* genome, and 6 μM Histone A monomer. Reagents for both tubes were diluted in Integration Buffer (20 mM PIPES pH 7.5, 200 mM KCl, 1 mM MnCl₂, 1% glycerol, and 10 mM TCEP) and incubated in a thermocycler at 40°C for 10 min. After this incubation, the contents of both tubes were rapidly combined to achieve final concentrations of 125 nM Pfu integrase (Cas1_4_-Cas2_2_-Cas4^11^), 3 μM Histone A, 25 nM foreign DNA, and 25 nM PfuCR7 520 leader. The combined reaction was incubated at 70°C for 5 min. The reaction was stopped by adding urea to a final concentration of 4 M, followed by incubation at 100°C for 15 min. The sample was then mixed 1:1 with 2× formamide loading buffer (95% formamide, 20 mM EDTA, 0.05% bromophenol blue, and 0.05% xylene cyanol) and denatured at 95°C for 15 min. Samples were resolved on a 7% (w/v) (29:1 mono:bis) polyacrylamide urea gel in 1× TBE (100 mM Tris-borate pH 8.3, 2 mM EDTA). Gels were washed with deionized water and imaged before and after staining with SYBR Safe (Invitrogen) using a LICORbio imager (Odyssey M) with detection channels “488” (excitation 488 nm, emission 519–543 nm), “700” (exc. 685 nm, emi. 721–740 nm), and “800” (exc. 785 nm, emi. 816–840 nm). The intensities of unintegrated, leader-side, spacer-side, and non-specific integration bands were quantified using Empiria Studio (LICORbio). These readings were used to calculate leader- and spacer-side integration events as percentages of all events. Full images of all gels are shown in the supplement.

### Histone binding assays

Binding reactions were assembled on ice in a buffer consisting of 20 mM PIPES pH 7, 200 mM KCl, 1 mM MnCl₂, 6.5% glycerol, and 10 mM TCEP. 0–1.1 µM of Histone A was titrated onto three different substrates: (i) 5 nM of a PCR product consisting of 520 basepairs of leader and the first two repeats of the CRISPR encoded adjacent to Cas2 in *P. furiosus*, (ii) 35 nM of a PCR product consisting of 91 basepairs centered on the proximal histone tetramer binding site, and (iii) 35 nM of a similar 91 basepair PCR product that contains the “h” mutation to the T2 motif which ablates CRISPR integration at the first repeat (Supplementary Table S1 and Supplementary Fig. S2). For gel-shift assays with DNA substrate (i), reactions were loaded onto a pre-run 2% agarose gel in TAE buffer (40 mM Tris base, 20 mM acetic acid, and 1 mM EDTA), and run at 150 V for 2.5 h at 4°C. Whereas for gel-shift assays with DNA substrates (ii) and (iii), reactions were loaded onto a pre-run 15% native polyacrylamide gel, and run at 150 V for 2 h at 4°C. All gels were stained using SYBR Safe DNA Gel Stain (Invitrogen, Thermo Fisher Scientific), and imaged on a LICORbio imager (Odyssey M) using detection channel “488” (excitation 488 nm, emission 519–543 nm).

For gel-shift assays with DNA substrates (ii) and (iii), which contain histone tetramer binding sites [22], the intensities of unbound and bound bands were quantified using Empiria Studio and used to calculate the fraction of bound DNA. Data analysis was performed with GraphPad Prism 10 software using nonlinear regression, specific binding with Hill slope, and least squares fit to calculate an apparent dissociation constant (*K*_d,app_) from three independent assays:


\begin{eqnarray*}
{\mathrm{Y}} = {{B}_{max}}{\mathrm{*}}\frac{{{{X}^h}}}{{K_d^h{\mathrm{\ }} + {\mathrm{\ }}{{X}^h}}}
\end{eqnarray*}

where *B*_max_ is the maximum fraction bound, *h* is the Hill slope, *K*_d_ is the concentration of Histone A to achieve half-maximum binding, *Y* is fraction of bound CRISPR leader, and *X* is Histone A concentration.

### *De novo* leader motif identification in euryarchaeal CRISPRs homologous to *P. furiosus* CRISPR–Cas

To collect and curate a set of CRISPR leaders homologous to those found in *P. furiosus*, we first used *P. furiosus* DSM 3638 sequences of Cas1, Cas2, Cas4^1^, Cas4^2^, and Histone A proteins (Uniprot IDs: Q8U1T7, Q8U1T8, Q8U1T6, Q8U027, and P61882, respectively) as queries for protein BLAST of the NCBI database. Genomes that had close homologs of all five proteins were found to be members of the taxonomic orders *Thermococcales* and *Methanococcales* (Supplementary Fig. S3), coinciding with the previously reported close evolutionary relationship of these two orders that form a distinct phylogenetic branch within the phylum Euryarchaeota [30]. Therefore, we downloaded 189 assemblies corresponding to these orders (131 *Thermococcales* and 58 *Methanococcales*) from the NCBI RefSeq Assembly database (accessed on 21 June 2025). CRISPR loci were identified in assemblies using a modified local copy of CRISPRDetect v3.0 that contains an updated list of known repeats, and with -T 0 -wgs 0 -annotate_cas_genes 1 and otherwise default parameters [23]. The default parameters exclude small CRISPR loci with aberrant spacers, such as a three repeat-long locus that is sometimes reported in the *P. furiosus* genome which contains one aberrant 57 bp-long spacer and a 37 bp-long spacer. The CRISPRDetect-based search resulted in the identification of 605 CRISPR loci from *Thermococcales* and 248 from *Methanococcales*. Most Archaeal CRISPR leaders have been reported to be <500 bps long, and the length of non-coding DNA upstream of most *P. furiosus* CRISPR loci is 480–500 bps (Fig. 2) [9]. Therefore, we extracted 500 bps of upstream DNA for all CRISPRs, using the CRISPRLeaderExtractor script (https://github.com/mbuyukyoruk/CRISPRLeaderExtractor). Any sequences for which 500 basepairs of upstream DNA were not available, for example because the CRISPR was on the edge of a contig, were removed at this stage. A non-redundant list of CRISPR leaders was generated by CD-HIT v4.8.1, using a 95% identity cutoff [31]. This cutoff was empirically determined through an iterative process to optimize the identification of informative motifs that did not overfit to redundant sequences. These curation steps resulted in a list of 349 *Thermococcales* and 193 *Methanococcales* leaders. CRISPR leaders from *Thermococcales* and *Methanococcales* were separately analyzed using a local copy of STREME (Simple, Thorough, Rapid, Enriched Motif Elicitation) [32], with the following parameters: streme -p *leader_sequences.fasta* -dna -oc *output_path* -bfile *background_file*-minw 5 -maxw 30. The *background_file* was generated with a second-order Markov model, using the following command: fasta-get-markov -dna -nostatus -m 2 *leader_sequences.fasta* > *output_file*. STREME analysis resulted in the discovery of 22 significant motifs in *Thermococcales* leaders and 14 in *Methanococcales* leaders. To determine if the motifs discovered for these two groups of leaders were similar to each other, known prokaryotic DNA sequence motifs [33–36], or motifs previously reported in Euryarchaeal CRISPR leaders [23], a local copy of the Tomtom Motif Comparison Tool was used with default parameters [37].

**Figure 2.**
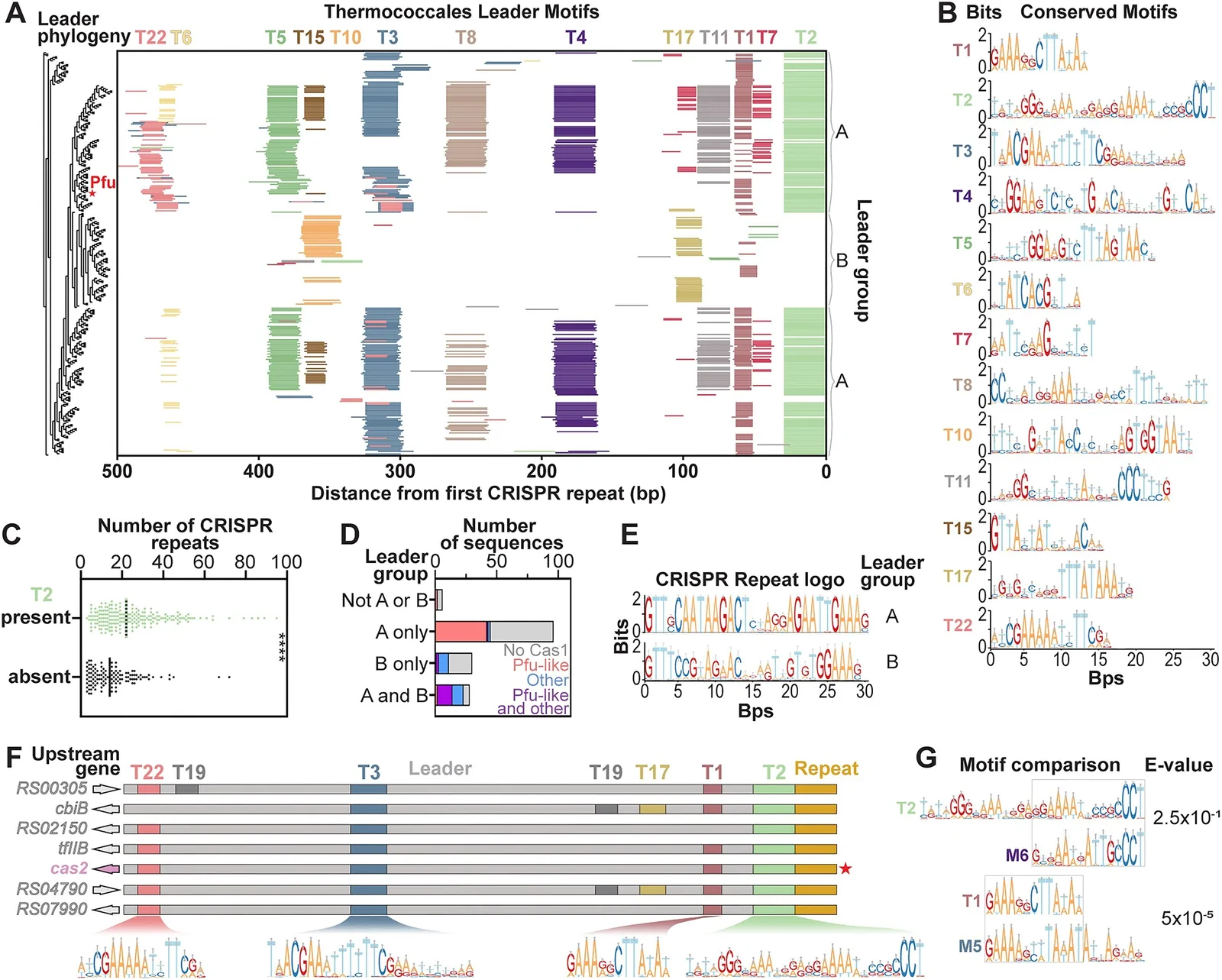
Thermococccales leaders contain conserved motifs up to 500 bps upstream of the CRISPR. (**A**) A phylogenetic tree generated from the alignment of 500 bps of CRISPR leader and the first repeat from 306 non-redundant sequences. 43 motif-less leaders were not included in this plot. The location of the CRISPR leader sequence from *P. furiosus* used in biochemical assays is indicated with a star. The CRISPR leader corresponding to each terminal node of the tree is represented in the plot as a horizontal line (not shown) extending from the node, and significant conserved motif occurrences are plotted as colored bars. Different motifs are depicted by unique colors and numbered from most significant (T1) to least (T22). Motif colors and numbers are shown above the plot. For clarity, only motifs that occurred in at least 10% of leaders are shown here (Supplementary Data S1 and S2). (**B**) Sequence logos of each motif. (**C**) The number of repeats in Thermococcales CRISPR loci downstream of leaders in which motif T2 is present or absent (Supplementary Data S3). The median is shown as a line. ^****^P < 0.0001, unequal variances *t*-test. (**D**) Comparison of the type of leaders and Cas1s encoded within the same Thermococcales reference sequence (Supplementary Data S4). For this analysis Group A leaders were defined as containing at least two of the following motifs: T1, T2, or T3. Group B leaders were defined as containing motif T10 or T17, or as lacking both T2 and T3. (**E**) Sequence logo of CRISPR repeats associated with Group A and Group B leaders. Repeat sequences are distinct in sequence and in length between these two groups. Repeats associated with Group A leaders were predominantly 30 bps in length (197/204 repeats = 97%), whereas repeats found downstream of Group B leaders were predominantly 29 bps long (74/118 = 63%) and had a greater variation in length. The representative repeats for a CRISPR locus downstream of a given leader, as determined by CRISPRDetect, were collated and separated by size prior to building a sequence logo. A sequence logo for the largest collection of similarly sized repeats was built using Weblogo v3.7.1 [38, 39]. (**F**) Schemes depicting the distribution of identified motifs across the seven CRISPR loci found in the *P. furiosus* genome. Logos for the four most highly conserved motifs are shown. The CRISPR leader used in biochemical assays is indicated with a red star. (**G**) Comparison of two sets of motifs of interest from CRISPR leaders found in *Thermococcales* (T2 and T1) and *Methanococcales* (M6 and M5). Motifs were compared with Tomtom [37] and were considered significantly similar if *E*-value is <10^−3^ (Supplementary Data S5).

To identify significant sequence matches to motifs in CRISPR leaders, a local copy of FIMO (version 5.5.8) was used with the Position Weight Matrices (PWMs) of STREME-detected motifs and the following parameters: fimo –oc *output_file* –verbosity 1 –no-pgc –bgfile *background_file* –thresh 1.0E-4 *streme_motifs.txt leader_sequences.fasta*. Then, to determine which motif occurrences were significant, a unique threshold for each motif was calculated based on the mean of the three most significant *q*-values for the motif occurrence in three sets of shuffled sequences. Shuffled sequences were created using the following command: fasta-shuffle-letters -kmer 1 -dna *leader_sequences.fasta shuffled_sequences.fasta*. A background file was generated for each set of sequences using a second-order Markov model. The lowest *q*-value for each motif occurrence in the shuffled sequences was obtained with FIMO using parameters: fimo –oc *output_path* –verbosity 1 –no-pgc –bgfile *background_file* –thresh 1.0E-2 *streme_motifs.txt shuffled_sequences.fasta*. The significance threshold for each individual motif was set as 100-fold lower than the mean lowest *q*-value for the motif occurrence in the shuffled sequences. Sequences of the leader and first repeat were aligned using MAFFT webserver (version 7) with iterative refinement alignment strategy (FFT-NS-i) and otherwise default parameters. Then, the IQ-Tree web server was used to create a phylogenetic tree using default parameters. Significant motif occurrences were plotted using start and end coordinates determined by FIMO. Sequence logos of the identified motifs were made using a FASTA file of the FIMO-identified matched sequences as input for Weblogo v3.7.1 [38, 39], using the following command weblogo -D fasta -F pdf -A dna -s large -n 100 –color “#D7191C” G “Guanine” –color “#FDAE61” A “Adenine” –color “#ABD9E9” T “Thymine” –color “#2C7BB6” C “Cytosine” –debug NO < *input.fa* > *output.pdf*.

## Results

### *P. furiosus* histones stimulate specific integration at the first repeat in a CRISPR array

Histone-mediated integration by the PfuCas1-2 complex has been previously reconstituted *in vitro*, with PCR amplification used to measure the relative amounts of rare integration events [22]. We sought to quantitatively measure histone-mediated integration, and to determine if Cas4 increases integration efficiency. To that end, we heterologously expressed and purified Histone A, Cas1, Cas2, and the Cas4 homolog (Cas4^1^) responsible for trimming the PAM, and a Cas4 homolog (Cas4^2^) that trims the 3′ end of the non-PAM end of the prespacer at a permissive NW-3′ recognition sequence [7].

We first performed a histone binding assay to determine the affinity for a linear CRISPR fragment consisting of 520 bps of upstream DNA, two CRISPR repeats, and one and a half spacers, which shows that DNA binding saturates at 0.8 µM of histone monomer (Supplementary Fig. S2). A histone concentration of 3 µM monomer was used for all integration assays to ensure the histone binding sites were saturated. We quantified histone-mediated Cas-catalyzed integration of a short DNA duplex that is fluorescently labeled on the 5′ ends (“prespacer”), into a linear CRISPR fragment that is truncated to 482 basepairs of upstream DNA, but which is otherwise the same as the CRISPR fragment used in histone binding assays. Consistent with previously published data, these integration reactions show that *P. furiosus* Cas1–Cas2 is sufficient for integration of the prespacer into the first CRISPR repeat (Fig. 1B,C and Supplementary Fig. S4) [22, 40].

To our surprise, we also find that Cas1 alone can bind the 3′ end of the non-PAM side of the prespacer and catalyze integration at the leader-repeat junction, when histone is present (Fig. 1B,C). We are aware of one previous report in which a similar control reaction was performed in the presence of a host factor (IHF), which did not observe the Type I-E Cas1 protein catalyzing a similar reaction [41]. This difference could suggest a divergence in the stability with which Cas1 is recruited by host factor-folded leader across these two systems [13].

The addition of Histone A decreases integration at off-target sites and simultaneously increases specific integration at the first CRISPR repeat by 1.4–2.7-fold depending on the concentration of Histone A and on the absence or presence of Cas4^1^ (Fig. 1C and Supplementary Fig. S5) [22]. A titration of Histone A reveals that a maximum increase in site-specific integration at the first CRISPR repeat (∼2.7-fold) is achieved by the addition of 1 µM of Histone A monomer (Supplementary Fig. S5). This assay further shows that up to 4.5 µM of Histone A is needed to minimize non-specific integration (∼1.4-fold), but such high concentrations of Histone A also decrease site-specific integration (Supplementary Fig. S5).

Trimming of the PAM is thought to be carefully coordinated to only occur after the Cas1-2 integrase has catalyzed transesterification of the 3′ hydroxyl of the non-PAM strand of the prespacer at the “leader”-side of the repeat [7, 25, 42, 43]. Therefore, Cas4^1^ must be associated with the Cas1-2–prespacer complex when the integrase localizes to the first CRISPR repeat. We hypothesized that Cas4^1^ might play a role in interacting with Histone protein or with CRISPR leader motifs to localize the integrase to the first repeat. However, Cas4^1^ did not increase integration efficiency over a range of Cas4^1^:Cas1 ratios and did not measurably trim the PAM from the prespacer over the course of this experiment (Fig. 1C and Supplementary Fig. S5). High concentrations of Cas4^1^ inhibited integration. For subsequent experiments, we chose to use a biologically relevant ratio of one Cas4^1^ to two Cas1 homodimers, which does not significantly reduce *in vitro* integration activity.

Conversely, Cas4^2^ purified under similar conditions as Cas4^1^ is a highly active and promiscuous nuclease that cleaves the prespacer at several locations (Fig. 1D and Supplementary Fig. S5), consistent with its previously determined permissive cleavage preference *in vivo* at “NW” dinucleotides (where N is any nucleotide, and W is A or T) [7]. Incubation of either Cas4^2^ or DNase I with fluorescently labeled prespacer generates fluorophore-labeled cleavage products that migrate slower through the polyacrylamide gel than the full-length prespacer strands (Supplementary Figs S5–S8). It is possible that the migration of very short fluorophore-labeled products could be influenced by the mass and charge of the fluorophore itself and migrate as if it were a large nucleic acid. As a reference, the commonly used gel-tracking dye Bromophenol blue has a molecular weight of 670 Daltons, but migrates through an agarose gel at the same rate as a 300 bp fragment of DNA, which weighs 195 000 Daltons [44].

The addition of Cas4^2^ in combination with Cas1, Cas2, Cas4^1^, and Histone A causes strands with extended 3′ ends to be frequently trimmed to a Cas1-2 integrase-protected fragment species (Fig. 1D, and Supplementary Figs S6 and S7). As such, Cas4^2^ trims the extended 3′ end of the PAM-containing prespacer strand which permits spacer-side integration (Fig. 1B,D). Cas4^2^ trims the prespacer prior to integration, causing both strands to be integrated at the leader-side and spacer-side of the CRISPR repeat. These data suggest that a condition, co-factor, or conformational change required to activate Cas4^1^ may be lacking. Overall, these results show that histones promote integration of DNA by the Cas integrase complex, and suggest that Cas1 or Cas2 may be sufficient to recognize the sequences in the CRISPR leader in a histone-dependent manner.

### Identification of conserved motifs up to 500 basepairs upstream of Euryarchaeal CRISPR leaders

DNA sequences ranging from 10 to 400 bps upstream of the CRISPR have been shown to promote integration of new spacers at the first repeat across diverse bacteria and archaea [4, 12, 14, 45, 46]. Sequence motifs that regulate integration are often strongly conserved across hundreds of related CRISPR leaders [14, 23]. To determine whether there are conserved DNA motifs upstream of the *P. furiosus* CRISPR that may regulate integration, we first searched the NCBI database with BLASTp to identify genomes containing close homologs of *P. furiosus* Cas1, Cas2, Cas4^1^, Cas4^2^, and Histone A. This search identified homologous type I-B Cas integrases and histones in two archaeal taxonomic orders, *Thermococcales*, of which *P. furiosus* is a member, and *Methanococcales* (Supplementary Fig. S3). Therefore, we downloaded all 189 genomic assemblies originating from the archaeal taxonomic orders *Thermococcales* and *Methanococcales*, including those that did not appear in the original BLASTp search. From these assemblies, we identified and curated a list of 349 *Thermococcales* and 193 *Methanococcales* non-redundant CRISPR loci. Specifically, a sequence identity cutoff of 95% was used to yield a diverse set of sequences suitable for motif discovery. This 95% cut-off ensures that no two sequences share >475 identical positions within a 500 bp leader. We then performed a *de novo* DNA sequence motif search using a combination of STREME (Simple, Thorough, Rapid, Enriched Motif Elicitation) and FIMO (Find Individual Motif Occurrences) [32, 47].

This analysis identified 36 motifs in total. Of these, 13 of the 22 *Thermococcales* motifs are found in at least 10% of 349 non-redundant *Thermococcales* leaders, and 9 of the 14 *Methanococcales* motifs are found in at least 10% of 193 non-redundant *Methanococcales* leaders (Fig. 2 and Supplementary Fig. S9). Motifs are numbered from most significant to least significant (e.g. motifs T1 to T22 for Thermococcales, and M1 to M14 for *Methanococcales*) according to STREME’s calculations (Supplementary Data S1 and S2) [32]. *Thermococcales* leaders often contained motifs at conserved positions up to roughly 480 bps upstream of the first repeat (Fig. 2). Whereas most *Methanococcales* leaders are shorter, such that conserved motifs were frequently found up to only 300 bps upstream of the first repeat (Supplementary Fig. S9). To determine whether the motifs we identified match any previously described archaeal protein binding sites or DNA motifs, we used the Tomtom algorithm to search for significant matches of the motifs identified here to databases of prokaryotic DNA motifs and to previously identified motifs in Archaeal leaders, and we manually inspected motifs (Supplementary Fig. S10) [23, 33–37] *Thermococcales* motif T1 and *Methanococcales* motif M5 strongly match to BRE and TATA boxes commonly found in archaeal transcriptional promoters [48–53]. No other motifs could be ascribed a biological function at this stage, despite their strong conservation in CRISPR leaders.

This analysis identified two predominant types of *Thermococcales* leaders. Roughly two-thirds of leaders (group A) contain three highly prevalent core motifs (e.g. motifs T1, T2, and T3) and several ancillary motifs (e.g. motifs T4–T8, T11, T15, and T22). Whereas approximately one-third of *Thermococcales* leaders (group B) are relatively sparse and predominantly contain only two motifs, annotated as T10 and T17 (Fig. 2A,B). Notably, group B leaders lack a strong BRE site adjacent to the TATA box (such as that seen in motif T1), but instead have a TATA box with a weak or degenerated BRE site (motif T17) (Fig. 2B) [53]. Intriguingly, motif T17 could be an example of an archaeal promoter that substitutes the canonical BRE site typically needed for the formation of the transcription pre-initiation complex with another factor [54]. The two types of *Thermococcales* leaders often co-occur in the same genome, and many *Thermococcales* genomes contain more than one CRISPR system and associated Cas integrase. Many bacteria and archaea with a single CRISPR system and integrase also possess multiple CRISPR loci which have been observed to accumulate new spacers at different rates, and to thereby accumulate fewer or greater numbers of spacers [14, 55–58]. T2 is the most prevalent motif amongst *Thermococcales* leaders, and it overlaps with one of the previously mapped histone binding sites thought to be needed for efficient integration [22]. Therefore, we hypothesized that the presence of motif T2 might denote highly active CRISPR loci. To test whether the presence of motif T2 denotes CRISPR loci that acquire and accumulate more spacers, we counted the number of CRISPR repeats in loci found downstream of leaders either containing or lacking motif T2. This analysis reveals that loci downstream of leaders that contain motif T2 tend to be larger than those that lack motif T2, possessing a median number of 22 and 14 repeats respectively (Fig. 2C and Supplementary Data S3).

Alternatively, we wondered whether the two distinct *Thermococcales* leader architectures observed correspond to two distinct integration mechanisms by a minimum of two different Cas integrases. Indeed, Cas1 found in reference sequences containing group A leaders were typically associated with close homologs of PfuCas1 (>55% sequence identity), whereas those with group B leaders were more likely to encode distant homologs of PfuCas1 (<36% sequence identity), and those with both types of leaders commonly encoded multiple Cas1 proteins (Fig. 2D and Supplementary Data S4). In 79 of the 160 *Thermococcales* reference sequences that contained CRISPR loci, no Cas1 could be identified by PADLOC [59]. Repeat sequences often co-vary with Cas1 protein sequences in a subtype-dependent manner [15, 60, 61]. A sequence logo of the representative repeat from CRISPR loci downstream of group “A” leaders and group “B” leaders reveals differences in the length (30 bps versus 29 bps) and sequence of the associated repeats (Fig. 2E). Collectively, these analyses suggest that the group “A” leaders facilitate efficient integration into group “A” repeats by Pfu-like Cas integrases to generate large CRISPR loci in these archaea.

*Methanococcales* leaders are comparatively more homogeneous than those from *Thermococcales* (Supplementary Fig. S9). Most *Methanococcales* leaders contain five conserved DNA motifs, which includes a motif that describes a BRE and TATA box (motif M5). Most of these leaders also contain a repeat-adjacent motif (M6), that bears some sequence similarity to the repeat-adjacent motif that appears in *Thermococcales* leaders (T2), although Tomtom did not report a statistically significant match (−log *E*-value ≥ 3) (Fig. 2G and Supplementary Fig. S10). Leaders from three strains of *Methanococcales voltae* were stark outliers, having only motif M13 (Supplementary Fig. S9).

Despite being found in different genetic contexts, upstream and downstream of different genes, all seven *P. furiosus* CRISPR leaders contain similar motifs up to nearly 500 bps upstream of the CRISPR (Fig. 2F). All *P. furiosus* leaders have significant sequence matches for motifs T2, which overlaps with the proximal histone binding site, and motif T3, which occurs 316 basepairs upstream of the CRISPR. Motifs T22 and T3 appear to be imperfect inverted repeats of each other, and they each contain imperfect palindromes (e.g. CGAAATTTTTTCG or CGAAAAATTTTCG, respectively) (Fig. 2 and Supplementary Fig. S10). Intriguingly, several bacterial CRISPR leaders have been previously reported to contain motifs that occur as inverted repeats of each other, and some of these play a role in CRISPR adaptation [14, 15]. Most of the *P. furiosus* CRISPR leaders also have confident matches to motif T1 which represents the BRE and TATA boxes, and motif T22, found 64 and 472 bps upstream of the CRISPR, respectively. Some of the leaders also contain matches to the less prevalent motifs T17 and T19. Collectively, the strong conservation of motifs T1, T2, T3, and T22 suggests they may play an important role in CRISPR biology.

### Histone binding sites and more than 302 bps of CRISPR leader DNA are required for efficient integration

Conserved leader motifs can have roles as DNA elements that regulate CRISPR transcription or integration, or as *cis-*acting RNA elements that regulate CRISPR RNA processing [11–15, 23, 62–69]. To determine whether the conserved motifs and histone binding sites in the *P. furiosus* CRISPR leader play a direct role in integration, we individually scrambled the sequence of each motif. These mutations preserve the GC-content of the segment but swap the order of nucleotides. The scrambled designs rendered the segments to no longer be confident matches to the original motif PWM (Position Weight Matrix), as determined by FIMO [47]. Mutation of distally located motifs T22 and T3 appears to have no impact on integration (Fig. 3B, lanes a and c), as the amount of integration achieved is within error of the wild-type (WT) sequence. Whereas scrambling the previously identified distal and middle histone binding sites modestly decreases integration activity by 1.3–1.4-fold, respectively, relative to WT (Fig. 3C) [22]. Conversely, scrambling a region that corresponds to the BRE and TATA boxes and partially overlaps with the proximal histone binding site increases integration efficiency by 1.3-fold (Fig. 3B, lane f). It is possible that the scrambled portion of the histone binding site is natively suboptimal to accommodate the partially overlapping transcriptional start site, and that the mutation does not disrupt histone binding or positioning. Strikingly, scrambling either the leader-portion of the histone binding site, or of just motif T2 (Fig. 3B, lanes g and h, respectively), ablates integration. Notably, scrambling motif T2 also ablates histone-independent integration at the first CRISPR repeat (Supplementary Fig. S11). Together, these data suggest that motif T2 may be a binding site for the Cas integrase complex.

**Figure 3.**
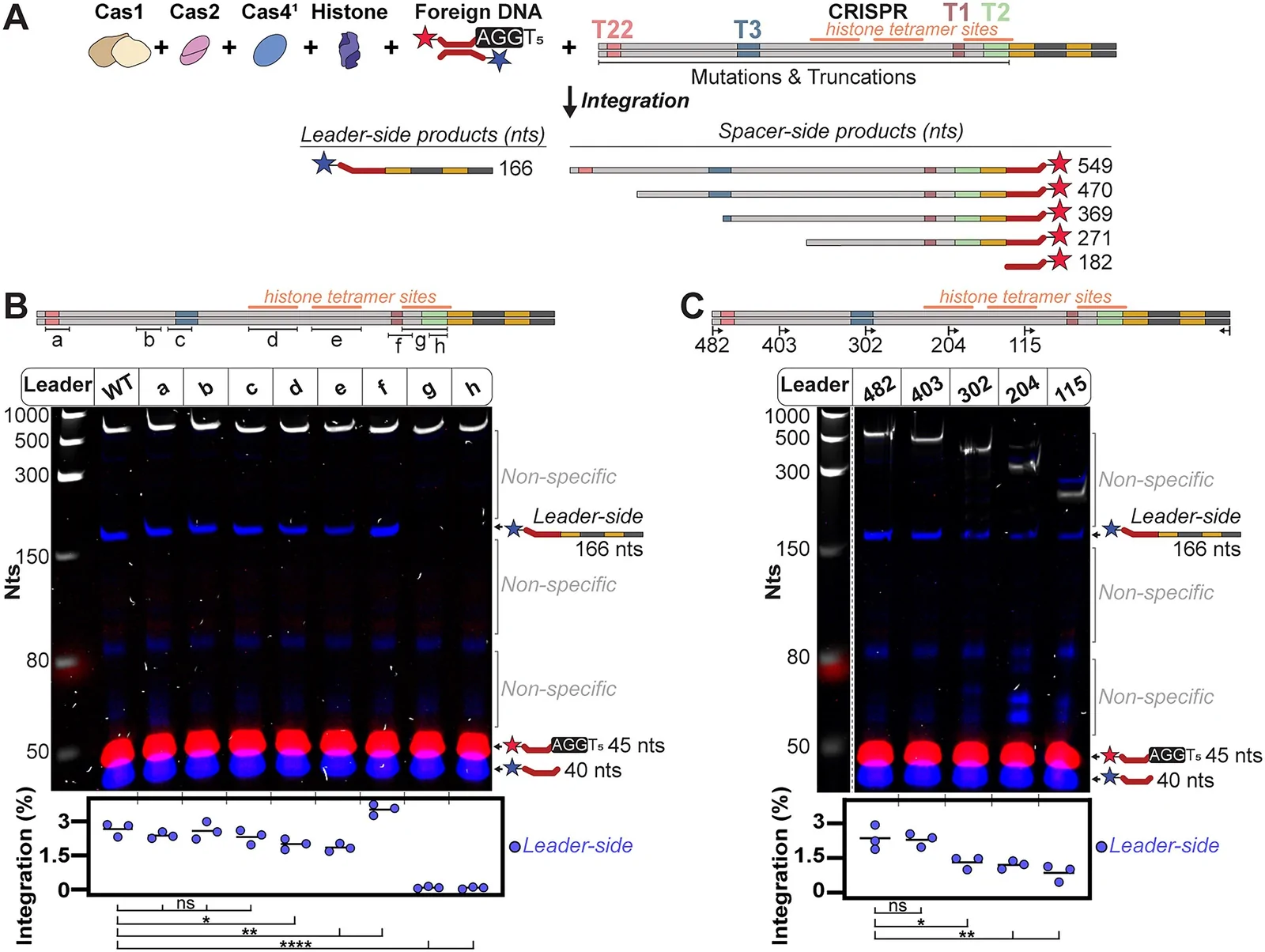
Up to 400 bps of *P. furiosus* leader facilitates histone-mediated CRISPR adaptation. (**A**) Scheme depicting substrates used to reconstitute CRISPR adaptation in a test tube, and expected integration products. The psPAM-5T prespacer used here consists of a strand containing the “AGG” PAM with a 5 nt-long thymidine overhang, and a non-PAM strand that has been trimmed. Truncations of the leader change the sizes of spacer-side integration products in an expected manner, shown. A different stock of purified Cas4^1^ was used in these assays shown in this figure as compared to Figs 1 and 4 (Supplementary Fig. S1). (**B**) Scheme of *P. furiosus* CRISPR substrate with scrambled regions denoted (top). Integration reactions with wild-type and scrambled leader variants resolved on a denaturing polyacrylamide gel (middle). Quantification of leader-side integration products from three independent reactions is shown below each corresponding lane (bottom). A representative of three replicate gels is shown (Supplementary Fig. S11). Mean and standard deviations are shown. Statistical significance was calculated using Dunnett’s multiple comparisons test, *P < 0.05,**P < 0.005,^****^P < 0.0001. (**C**) Scheme of *P. furiosus* CRISPR substrate with annotations denoting the 5′ ends of primers used to generate leader truncations (Supplementary Table S2) (top). Integration reactions with wild-type and truncated leader variants resolved on a denaturing polyacrylamide gel (middle). Quantification of leader-side integration products from three independent reactions is shown below each corresponding lane (bottom). A representative of three replicate gels is shown (Supplementary Fig. S12). Mean and standard deviations are shown. Statistical significance was calculated using Dunnett’s multiple comparisons test, *P < 0.05, **P < 0.005, ^****^P < 0.0001. The ladder and unlabeled initial CRISPR fragment substrate were visualized with SYBR Safe (white), the non-PAM strand is labeled with IRDye^®^ 700 (blue), and the PAM-containing strand is labeled with IRDye^®^ 800 (red).

Scrambling motifs is a direct method to test the impact of individual regions of interest in the context of a full-length substrate. However, the impact of these mutations on integration can be subtle even for sequences known to play a structural and mechanistic role in the assembly of the integration complex [14, 15]. To complement the motif scrambling assay with a more drastic perturbation, we sequentially truncated the leader by roughly 100 bp increments. In agreement with the scrambled motif results, a truncation that removes motif T22 does not affect integration efficiency compared to wild-type (Fig. 3C, truncation “403”). However, further truncation of the leader to 302 bps, which removes most of motif T3, decreases integration efficiency by 2-fold for integration of the psPAM-5T substrate relative to wild-type (Fig. 3A,C, and Supplementary Fig. S12). Further truncation of the leader to 204 bps, which halves the distal histone binding site, decreases leader-side integration of the psPAM-5T prespacer by 2.7-fold relative to wild-type. Whereas truncation of the leader to 115 bps, which removes most of the middle histone binding site, decreases integration efficiency by 3.6-fold relative to wild-type (Fig. 3C). New off-target integration events occur with the 115 bp leader construct, and likely correspond to integration at the edges of the target CRISPR DNA. Spacer-side integration exhibited a similar trend to leader-side integration and is only seen for the psPAM substrate, which contains a PAM but lacks a five thymidine-long extension 3′ to the PAM that is known to stabilize Cas4 binding (Supplementary Fig. S12) [42, 43]. Conversely, no spacer-side integration of the psPAM-5T substrate is seen. Further, we see no evidence of PAM processing from either substrate by Cas4^1^, and the PAM-containing strand is efficiently and erroneously integrated at the leader-side of the repeat (Figs 1C and 3C, and Supplementary Fig. S12). Therefore, it appears that, unlike the type I-F Cas integrase, the *P. furiosus* type I-B complex is promiscuous and will efficiently integrate a range of prespacer lengths in the absence of Cas4^2^  *in vitro*, as has been previously reported *in vivo* (Supplementary Fig. S6) [7, 15]. Overall, the integration results for both prespacer substrates on truncated leaders suggests that up to 403 basepairs of DNA upstream of the *P. furiosus* CRISPR loci is needed for efficient integration at the first repeat.

### T2 leader motif sections are required for histone-dependent and histone-independent integration

We were intrigued by the observation that scrambling the T2 motif inhibited both histone-dependent and histone-independent integration at the first CRISPR repeat (Fig. 3 and Supplementary Fig. S11). PfuCas1 has previously been reported to possess a preference for cytosine at the −3 bp position upstream of the integration site [40], which is consistent with the high conservation of cytosine that we observe in the T2 motif (Fig. 4A). Similarly, *E. coli* Cas1 has been shown to have an intrinsic preference for a specific nucleotide at the −2 position [70, 71]. Therefore, it is possible that the scrambles we designed (e.g. mutant “h”) mutated a single nucleotide that is directly recognized by PfuCas1, and which inhibits integration at a step that is downstream of histone binding or otherwise histone-facilitation of integration. To determine which regions of the T2 motif are critical for histone-dependent integration, histone-independent integration, or both, we measured integration activity into either CRISPR substrates containing transversion mutations tiled across the T2 motif, or a swap of the proximal histone binding site sequence with that of the middle histone binding site (Fig. 4A).

**Figure 4.**
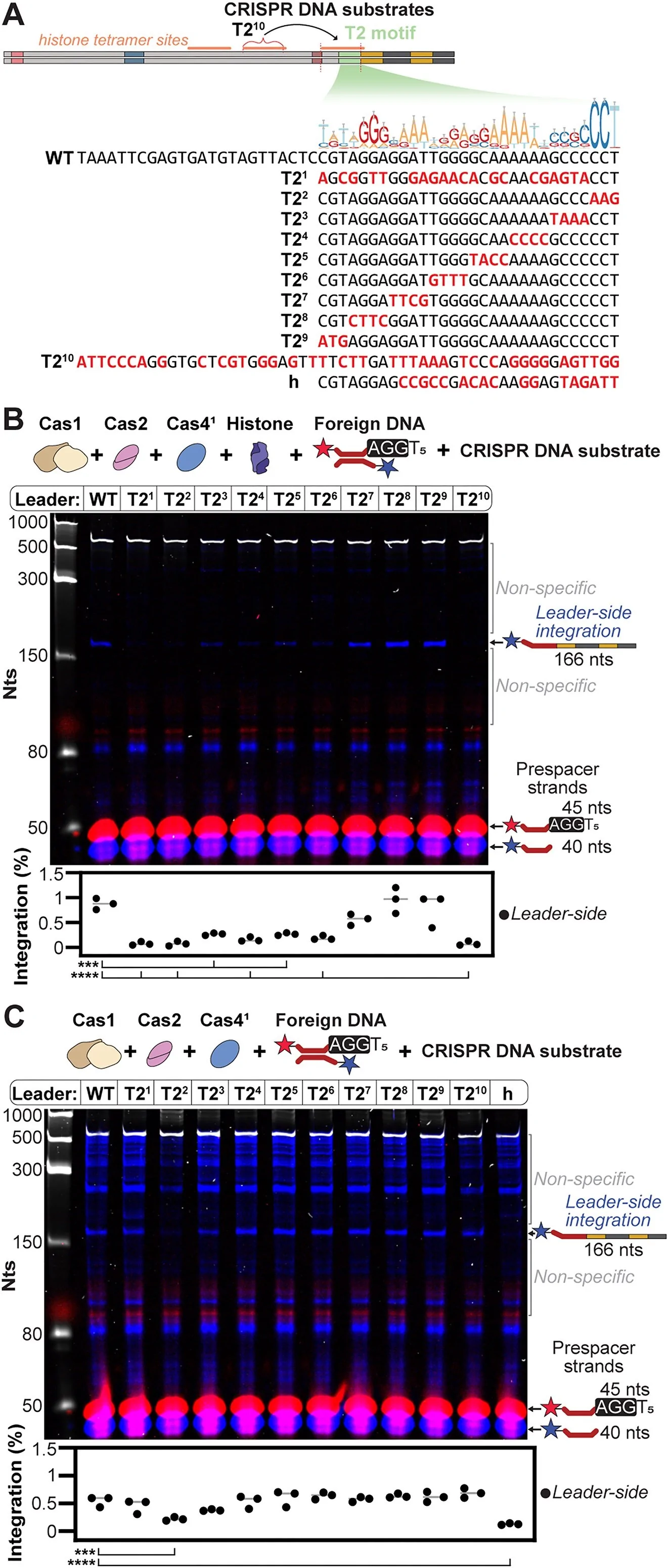
The T2 leader motif contains sequences required for histone-dependent and histone-independent integration. (**A**) Scheme of *P. furiosus* CRISPR substrate with mutated regions denoted. Most mutants possess transverse mutations of distinct regions of the T2 motif (mutants T2^1^-T2^9^), one mutant is a scramble of the T2 motif also examined in Fig. 3 (mutant h), and one mutant is a replacement of the proximal histone binding site (excluding the CRISPR repeat sequence) with a corresponding region from the middle histone binding site (mutant T2^10^). The proximal histone binding site extends into the first seven bps of the repeat. The seven bps of the repeat were left unchanged in mutant T2^10^ to preserve the integration target site. (**B**) Integration reactions with wild-type and mutant leader variants in the presence of Histone A, Cas1, Cas2, and Cas4^1^ resolved on a denaturing polyacrylamide gel (top). Quantification of leader-side integration products from three independent reactions is shown below each corresponding lane (bottom). A representative of three replicate gels is shown (Supplementary Fig. S13). (**C**) Histone-independent integration reactions with wild-type and mutant leader variants in the presence of Cas1, Cas2, and Cas4^1^ resolved on a denaturing polyacrylamide gel (top). Statistical significance was calculated using Dunnett’s multiple comparisons test, ***P < 0.0005,^****^P < 0.0001. For visual clarity, non-significant differences relative to WT are not annotated. The ladder and unlabeled initial CRISPR fragment substrate were visualized with SYBR Safe (white), the non-PAM strand is labeled with IRDye® 700 (blue), and the PAM-containing strand is labeled with IRDye® 800 (red).

A leader variant carrying transversion mutations of the first 3 basepairs of the leader (T2^2^) exhibits no histone-mediated integration at the first CRISPR repeat (Fig. 4B and Supplementary Fig. S13). Similarly, mutation of individual 4 bp-long windows that tile from position −4 to −19 of the leader greatly inhibit integration (T2^3^-T2^6^). Additionally, scrambling residues −4 to −30 drastically inhibits integration (T2^1^). Although mutation of a window containing basepairs −20 to −23 slightly decreases integration efficiency relative to wild-type, this difference is not statistically significant. Conversely, mutation of basepairs −24 to −27 or −28 to −30 have no effect on integration relative to wild-type.

We wondered whether histone binding is grossly affected by mutations that prohibit histone-mediated integration. Gel shift assays reveal that mutant “h”, which ablates integration (Fig. 3B), has a minor impact on histone affinity, causing the dissociation constant to increase from 540 to 590 nM (Supplementary Fig. S2). This decrease in affinity is unlikely to significantly decrease the amount of histone-bound leader in integration assays, in which 3 µM Histone A was used, though it may position the histones differently (Supplementary Fig. S2). Further, replacing the proximal histone binding site with the sequence of the middle histone binding site greatly decreases histone-mediated integration (mutant T2^10^) (Fig. 4B). Together these results suggest histone-binding alone is not sufficient for histone-mediated integration.

To distinguish whether T2 mutations affect histone-facilitated integration, or affect Cas1-catalyzed integration more directly, we also measured integration in the absence of Histone A (Fig. 4C). Mutants that preserve the wild-type sequence in basepairs −1 to −3, but otherwise change the sequence of basepairs −4 to −30 of the leader do not negatively impact histone-independent integration (e.g. T2^1^ and T2^3^-T2^9^). However, a transverse mutation of basepairs −1 to −3, “TCC” to “GAA,” ablates integration at the first repeat. Mutant “h,” which contains a transversion mutation at position −3, and a transition mutation at position −2, also inhibits histone-independent integration at the first CRISPR repeat (Fig. 4 and Supplementary Fig. S13). Whereas swapping the middle histone binding site for the proximal binding site (T2^10^), which introduces a transition mutation at position −3 and transversion mutations at positions −1 and −2 (along with 36 additional upstream mutations), does not inhibit histone-independent integration at the first CRISPR repeat.

Collectively these experiments reveal distinct roles for sections of the T2 motif, which encompasses the first 30 basepairs of the *P. furiosus* CRISPR leader. A pyrimidine at position −3 is critical for histone-dependent and histone-independent integration. Further, basepairs −4 to −19 are critical for histone-dependent integration but dispensable for histone-independent integration, and so may not be directly recognized by the Cas integrase. It is unlikely that basepairs −4 to −19 are required for tight histone binding, since scrambling the T2 motif has a minimal effect on histone affinity (Supplementary Fig. S2E). Finally, the wild-type sequence of positions −1, −2, and −20 to −30 of the leader are not necessary for integration.

## Discussion

Cas integrases have evolved to perform site-specific and directional integration of DNA, often via the guidance of extra DNA that has been bent into distinct shapes [13, 15]. Site-specific and directional integration are sought after enzymatic behaviours in gene editing applications; therefore, a mechanistic understanding of CRISPR integration may reveal insights helpful for such translational research. Here we demonstrate that archaeal histones cooperate with up to 403 basepairs of DNA upstream of the CRISPR to promote efficient and specific Cas-catalyzed integration at the first CRISPR repeat (Fig. 5). We identify a 30 bp DNA motif in *Thermococcales* CRISPR leaders, T2, that overlaps with the proximal histone binding site in *P. furiosus* CRISPR leaders and is critical for both histone-mediated and histone-independent integration at the first repeat (Figs 2 and 3). A series of mutations across the T2 motif suggest the −3 position of the leader must be a pyrimidine to support integration at the leader-repeat junction. This finding is consistent with a previous report that states the *P. furiosus* Cas integrase has a preference of cytidine at the −3 position relative to the site of integration [40]. Additionally, basepairs −4 to −19 of the leader are critical for histone-dependent integration. Scrambling 22 bps of the 30 bp T2 motif (mutant “h”) does not greatly perturb histone binding affinity (Supplementary Fig. S2). Therefore, the specific sequence of the T2 motif may be needed to either ensure proper placement of the histone tetramer at this critical juncture, or it may serve as a Cas integrase recruitment sequence in the context of histone-folded DNA but not in unfolded DNA.

**Figure 5.**
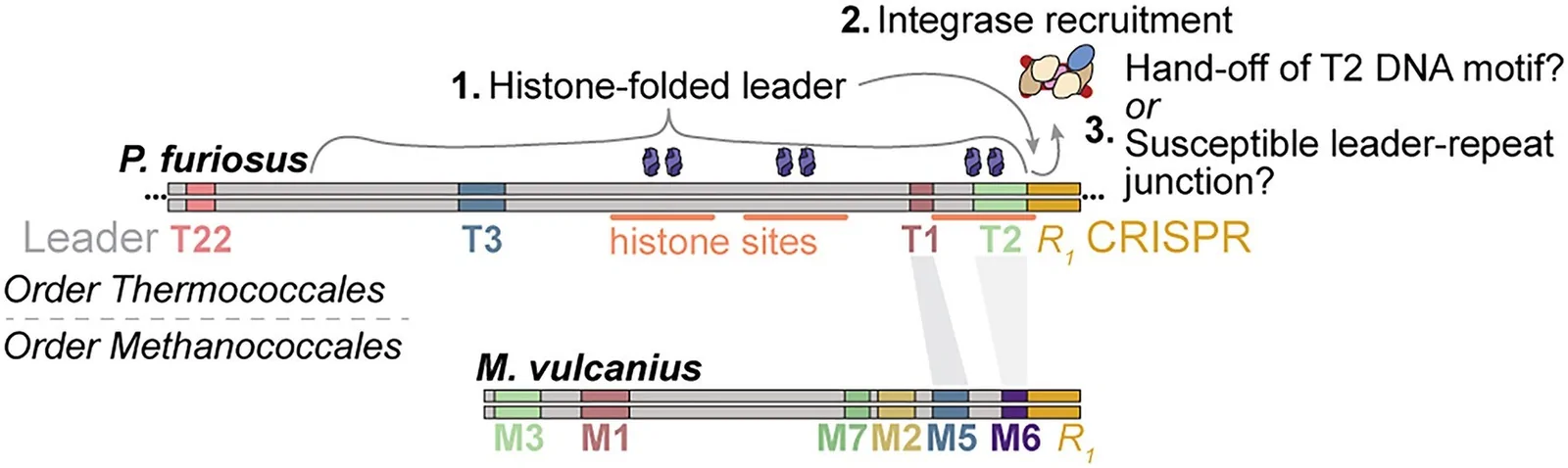
Histone-mediated CRISPR adaptation model. Most CRISPR loci in the order *Thermococcales* share leader motifs T22, T3, T1, and T2 found up to 500 basepairs upstream of the CRISPR. *P. furiosus* leaders tend to contain these four motifs and three histone binding sites. Up to 400 basepairs of leader, and three histone binding sites, facilitate efficient integration at the leader repeat junction. Histone-mediated folding of the leader likely forms this genomic locus into a shape that recruits prespacer-bound Cas integrase. The T2 DNA sequence may either be required for positioning the proximally bound histone tetramer, or for recruiting the Cas integrase complex in a histone-dependent manner. The last 7 bps of the proximal histone binding site overlap with the repeat sequence [22]. Therefore, there must either be a hand-off of the leader-repeat junction DNA from histone to Cas1, or histone and Cas1 must bind opposing surfaces of the DNA duplex at this junction for transesterification to proceed. If the latter is true, histone-induced DNA bending may in part promote integration by increasing accessibility of the leader-repeat junction for Cas1-catalyzed transesterification. Most CRISPR loci in the order *Methanococcales* contain leader motifs M3, M1, M7, M2, M5, and M6, found up to 300 basepairs upstream of the CRISPR. Motifs T1 and M5 are found in a similar location within the leader, share strong sequence identity (*E*-value = 5 × 10^−5^), and appear to represent a BRE-TATA box. Motifs T2 and M6 both adjoin the first CRISPR repeat and are similar in sequence (*E*-value = 2.5 × 10^−1^), though they are not significant matches to each other.

The T2 motif is found in all seven *P. furiosus* CRISPR leaders, and is strongly conserved across *Thermococcales* CRISPR leaders. Further, *Methanococcales* leaders contain a motif with a similar sequence that is also found adjacent to the first repeat (Fig. 5, and Supplementary Figs S9 and S10). Therefore, the T2 motif may be broadly important for efficient CRISPR adaptation across two taxonomic orders.

T3 is the only motif other than T1 and T2 that is conserved across all seven *P. furiosus* CRISPR leaders. Scrambling T3 did not appear to impact integration efficiency (Fig. 3B). However, truncation of the leader from 482 to 302 bps, which truncates T3, decreases integration efficiency by 2-fold (Fig. 3C). These data suggest that either partial truncation of this motif or removal of a yet-unidentified sequence found 403 to 302 bps upstream of the CRISPR affects integration efficiency. Notably, motif T3 is one of the top 3 most significant and prevalent motifs found in *Thermococcales* leaders (Fig. 2), alongside motif T1 which represents a sequence required for transcription initiation (the BRE and TATA boxes), and T2 which is required for integration (Figs 3 and 4). Therefore, motif T3 may represent another prevalent sequence with a critical role in CRISPR biology and integration.

Cas and non-Cas nucleases often facilitate trimming of the prespacer to an integrase-protected segment that is subsequently integrated into the CRISPR locus [7, 42, 72–74]. The ancillary CRISPR-associated nuclease Cas4 ensures prespacers contain a PAM sequence and are integrated in the correct orientation by Cas1-2 [4–8]. Cas4 binds to the PAM and occludes the corresponding 3′OH, favoring Cas1-catalyzed integration of the non-PAM 3′OH of the prespacer. In the I-G CRISPR system, interactions of the half-integrated CRISPR DNA with the Cas integrase have been proposed to stimulate the PAM-cleavage activity of Cas4, which is followed by Cas4 dissociation from the integrase, and subsequent integration of the second prespacer strand at the spacer-side of the CRISPR repeat [42]. *P. furiosus* encodes two Cas4 proteins, where Cas4^1^ conducts the canonical Cas4 role of specifying the PAM, and Cas4^2^ acts as a non-specific nuclease that trims the non-PAM end of prespacers [7]. In the absence of these Cas4 nucleases, Cas1-2 integrates longer prespacers and is not specific for DNA sequences adjacent to a PAM *in vivo* [7]. Similarly, we find that Cas1-2 can fully integrate prespacers *in vitro* that would generate a spacer three nucleotides longer than the typical spacer length of 37 bps (Supplementary Fig. S12). We show that Cas4^2^-based cleavage of prespacers is enhanced in the presence of Cas1-2, and Cas4^2^ tends to cleave both the non-PAM and the PAM-containing strands of the prespacer (Fig. 1 and Supplementary Figs S5–S8). Cas4^2^ trims the extended 3′ overhang and PAM from a long prespacer to license subsequent spacer-side integration (Fig. 1). Conversely, Cas4^1^ purified under similar anaerobic conditions, did not cleave the PAM under the conditions tested. Quantification of the iron content of Cas4^1^ suggests that most subunits (80%–89%) are loaded with a complete iron–sulfur cluster, therefore inactivity is likely not due to insufficient active protein (Supplementary Fig. S1). It is possible that an unidentified trigger or conformational change in the integrase complex may be required to stimulate Cas4^1^ activity after leader-side integration of the non-PAM strand has occurred.

The occupancy of the three histone tetramer binding sites found in *P. furiosus* CRISPR leaders does not clearly correlate with their relative importance in integration (Fig. 3B) [22]. A nuclease protection assay of genomic DNA isolated from *P. furiosus* has previously shown that the middle histone binding site is most protected from nuclease degradation, followed by the proximal site, and finally the distal site [22]. Here, we show that scrambling the middle and distal histone binding sites cause a modest decrease in integration, whereas mutating several separate 4-bp windows of the proximal histone binding site is sufficient to completely inhibit histone-mediated integration (Figs 3B and 4). Further, swapping the sequence of the leader-portion of the proximal histone binding site (which extends into the CRISPR repeat by 7 bps) does not generate a CRISPR leader that can sustain histone-mediated integration at the first CRISPR repeat (Fig. 4). Additionally, our search for conserved motifs failed to identify a sequence overlapping with the distal or middle histone binding sites, but confidently identified a conserved motif (T2) overlapping with the proximal histone binding site. Collectively, these data suggest that while all three histone tetramer binding sites are needed to bind and fold the leader into a shape that promotes integration at the first repeat, the proximal histone binding site has a unique role defined by a distinct DNA sequence.

Long-range DNA interactions from sequence motifs found up to 130 bps upstream have been visualized to promote site-specific CRISPR integration in bacteria [15]. The type I-F CRISPR leaders from *Pseudomonadota* bacteria contain proximal and distal binding sites for the DNA bending protein IHF [14, 15, 75], somewhat analogous to the proximal and distal histone binding sites found in *P. furiosus* type I-B CRISPR leaders. Structures have revealed that the distal IHF site inserts a U-bend in the leader that places two flanking inverted repeat motifs in 3-dimensional space to form parallel DNA pillars that recruit prespacer-bound type I-F Cas integrase, whereas the proximal IHF site is required to dock the first CRISPR repeat into the Cas1 active site [15, 76]. Expectedly, mutation of the proximal IHF binding site ablates integration [14], perhaps analogously to the effect of mutating the proximal histone binding site of the *P. furiosus* leader studied here (Figs 3 and 5). Strikingly, despite the critical structural role for IHF binding at the distal site, removal of this motif resulted in only a 2-fold decrease in integration efficiency [14]. Here we similarly find that proximal leader motifs tend to exhibit stronger effects in integration assays than distal motifs (Fig. 3).

Future structural analyses of a complete *P. furiosus* integration complex will reveal how histones bind to multiple sites along the leader to coordinate up to 403 bps of DNA to promote efficient CRISPR adaptative immunity, and how the T2 motif mediates histone-dependent integration. Intriguingly, some eukaryotic retroviruses that preferentially integrate at nucleosomal DNA sequences have evolved a mechanism to slide and lift DNA off histones to gain access to the phosphate backbone [77]. The type I-F Cas1 protein has been shown to contact the minor groove of bps −1 to −4 of the leader and + 1 of the repeat, and the major groove of bps +2 and +3 of the repeat, occluding one half of the DNA duplex at this junction [15]. Experimentally determined structures are required to unveil whether a hand-off mechanism is required to transfer the leader-repeat junction and motif T2 from histone to Cas integrase, or whether the histones bind the opposing face of the DNA duplex to induce a bend in the leader-repeat junction backbone that facilitates Cas1-catalyzed nucleophilic attack and subsequent integration of new prespacers.

## Supplementary Material

gkag1172_Supplemental_Files

## Data Availability

The data underlying this article, including full gel images, are available in the article and in its online supplementary material.

## Notes

Present address: Department of Biological Chemistry, University of Michigan, Ann Arbor, MI, 48109, United States
