# Supplementary material for: Archaeal histones coordinate conserved DNA sequence motifs to promote CRISPR adaptive immunity
Source: Nucleic Acids Res. 2026 Nov 10;54(21):gkag1172. doi: 10.1093/nar/gkag1172 (PMC13748974; doi:10.1093/nar/gkag1172)
Supplement: Supplementary file 1 [file gkag1172_supplemental_files.zip › Pfu_Supplemental_Figures_Revised_Fin.pdf]

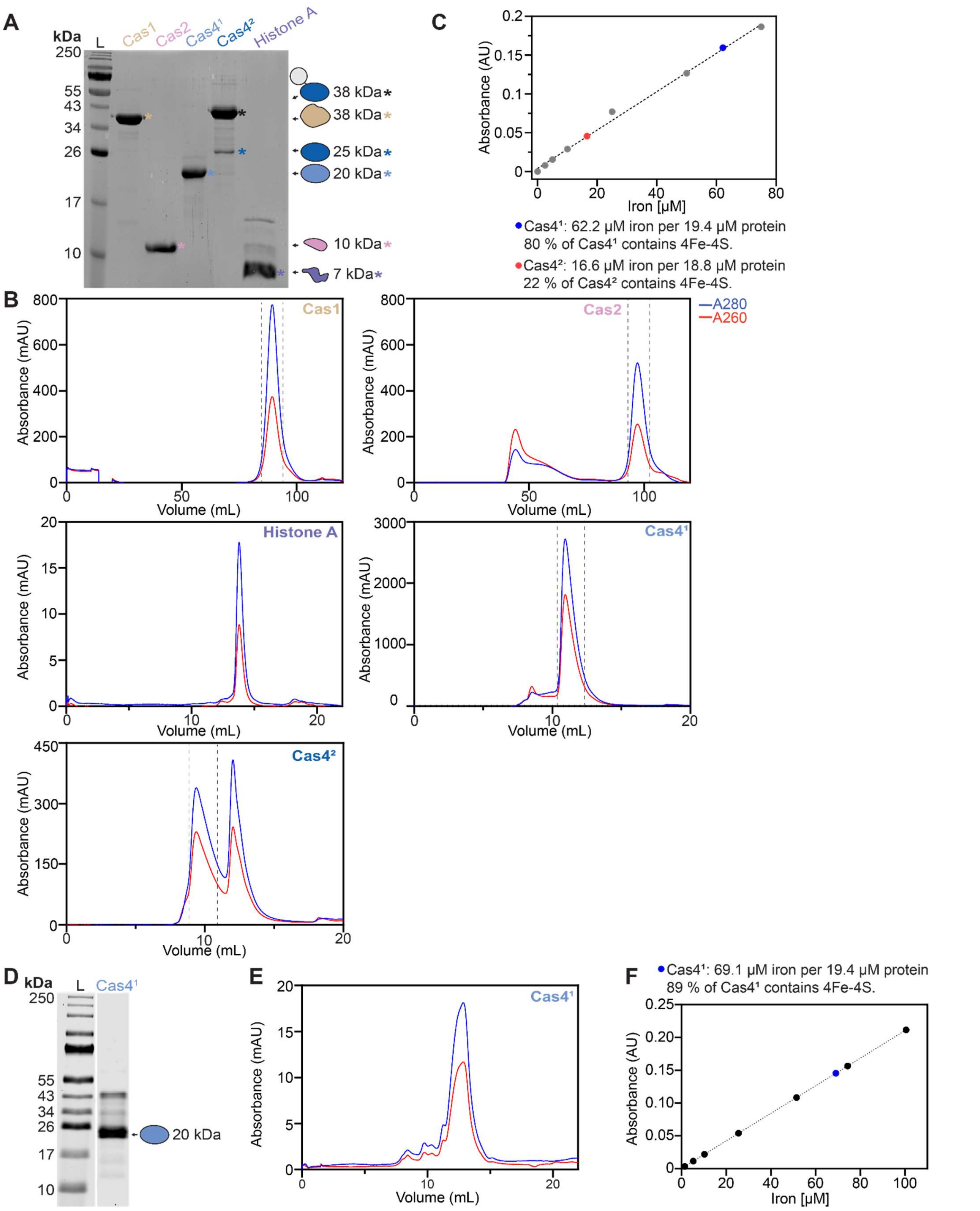

Supplemental Figure S1. Purification of *P. furiosus* Cas integrase and histone proteins. (**A**) Tricine SDS-PAGE gel of purified Cas1, Cas2, Histone A, Cas4[1] and Cas4[2] proteins. Cas2, Cas4[1] and Cas4[2] migrate slower than expected for their molecular weight. The bands corresponding to untagged protein are denoted with an

asterisk of the corresponding color. (**B**) Chromatograms of purified proteins. Fractions collated for the final protein stock are denoted between dotted lines. Protein within this region of the chromatograms were used for assays. For Histone A, aliquots of the final protein stock were re-run on size-exclusion chromatography columns. For Cas4^{2}, the tagged protein fractions had higher iron content and so were used for downstream biochemistry assays. (**C**) Ferrozine-based quantification of iron amount in Cas4^{1} and Cas4^{2} protein stocks, and estimation of the amount of Cas4 proteins loaded with a 4Fe-4S cluster [1]. The Cas4 protein stocks were assumed to be pure for this estimation, therefore the calculated amount of proteins loaded with a 4Fe-4S cluster is an underestimation. The Cas4^{1} characterized in panels A-C was used in all experiments except those depicted in Figure 3 and the corresponding Supplemental Figure S11. (**D**) SDS-PAGE gel of Cas4^{1} purified using a Heparin column in the anaerobic chamber (rather than SEC outside of the chamber) as the final polishing purification step (see Methods for additional details). This Cas4^{1} stock was used for experiments depicted in Figure 3 and the corresponding Supplemental Figure S11 and S12. (**E**) SEC Chromatogram of Cas4^{1} purified using a Heparin column as the final purification step. A fraction of the final prep was run on an SEC column. (**F**) Ferrozine-based quantification of iron amount in Cas4^{1} purified using a Heparin column as the final purification step.

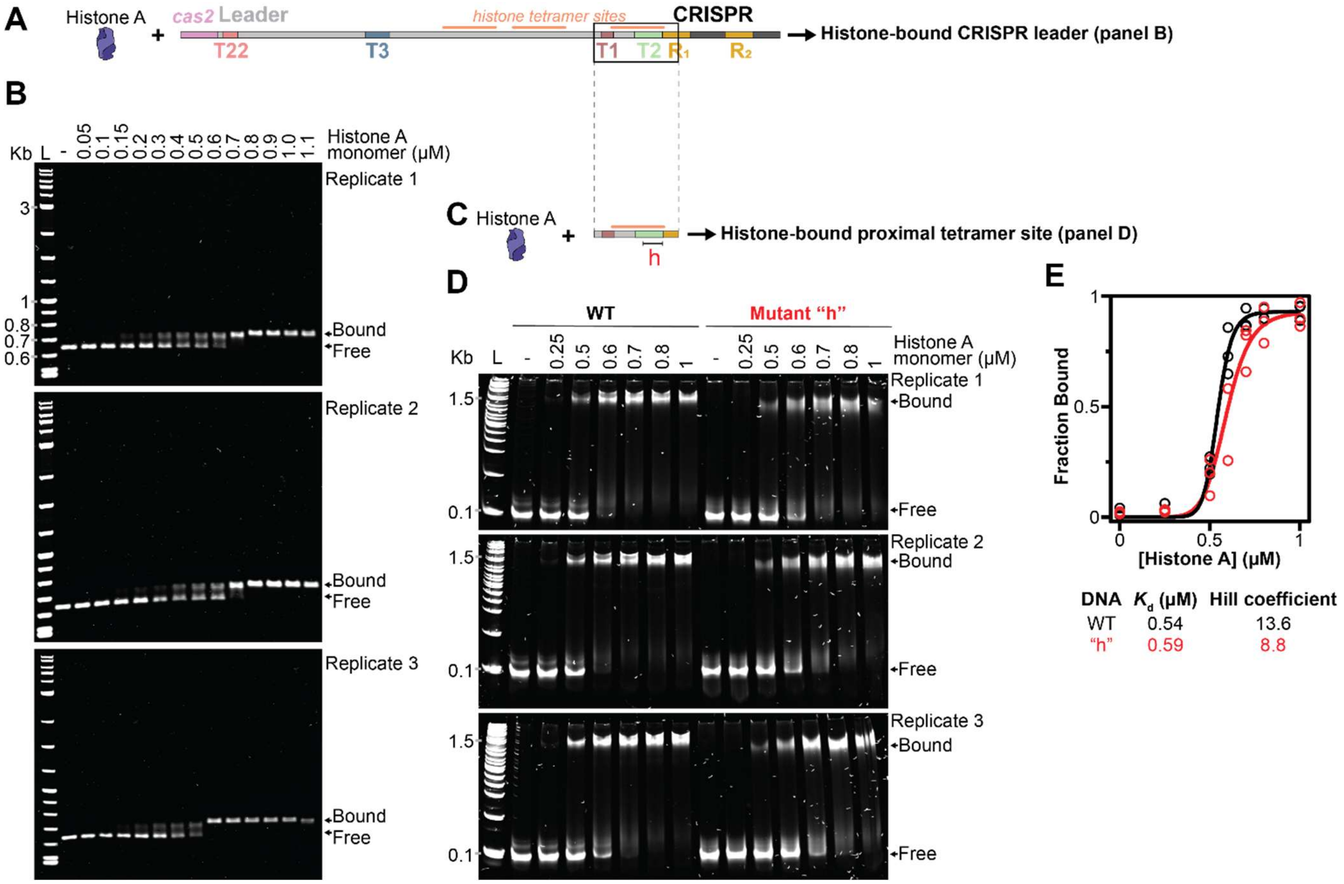

Supplemental Figure S2. Histone binding assays guide integration assay conditions and suggest mutations do not greatly inhibit binding affinity. (**A**) Scheme of histone binding assay to full-length CRISPR integration substrate. Histone A was titrated into a solution containing 5 nM of a double-stranded DNA fragment containing 520 bps of the leader, as well as two CRISPR repeats and two spacers. This DNA fragment is the wildtype CRISPR-containing sequence used in integration assays in this manuscript. (**B**) Images of three independent histone titration experiments, resolved on a native agarose gel. The gel was stained with SYBR Safe post-run. Histone A was titrated from 0-1.1 μM of monomer. (**C**) Scheme of histone binding assay to the proximal histone tetramer binding site. Double-stranded DNA fragment corresponding to a 91 bp sequence centered on the 60-61 bp proximal histone binding site was used as a substrate for a gel-shift assay. The substrate contained either the wildtype sequence (WT) or contained a mutation ("h") that ablates integration (**Figure 3**)[2]. (**D**) Images of three independent histone titration experiments, resolves on a native polyacrylamide gel. The gel was stained with SYBR Safe post-run. Histone A was titrated from 0-1 μM of monomer. (**E**) Best fit parameters for binding to WT and mutant proximal histone binding site reveals that the mutation has a minimal if any effect on binding affinity and decreases the Hill coefficient of binding.

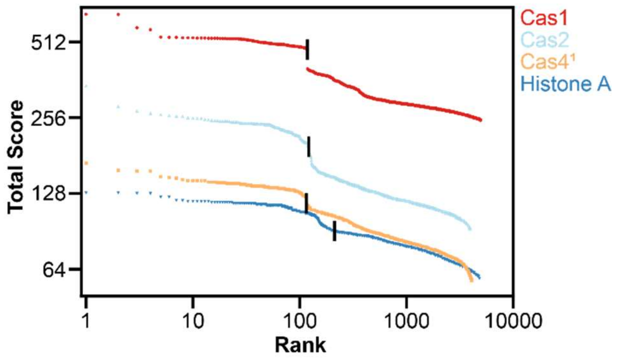

Supplementary Figure S3. BLAST-based Cas and Histone homolog search informs selection of archaeal genomes for homologous leader analysis. BLAST results of *Pyrococcus furiosus* Cas1, Cas2, Cas4-1, and Histone A from searching the NCBI non-redundant protein sequences database. BLAST hits were ranked in order from high to low Total Score. The x-axis shows the rank, and the y-axis shows the Total Score. Each point represents one protein hit for a given Cas or Histone protein. The scores of Cas and histone homologs were used to guide the selection of genomes analyzed for homologous CRISPR leader analysis (**Figure 2**). There is a steep drop-off of Total Score at the cutoffs chosen for Cas proteins, suggesting that proteins to the left of the cutoff (Rank 1-~100) are close homologs, whereas those to the right of the cutoff or distant homologs. Total Score cutoffs for Cas1 (cutoff=477), Cas2 (117), Cas4-1 (196), and Histone A (90.9) are indicated with a black bar. *Thermococcales* and *Methanococcales* were the only orders represented in hits at or above the respective Total Score cutoff across all four proteins.

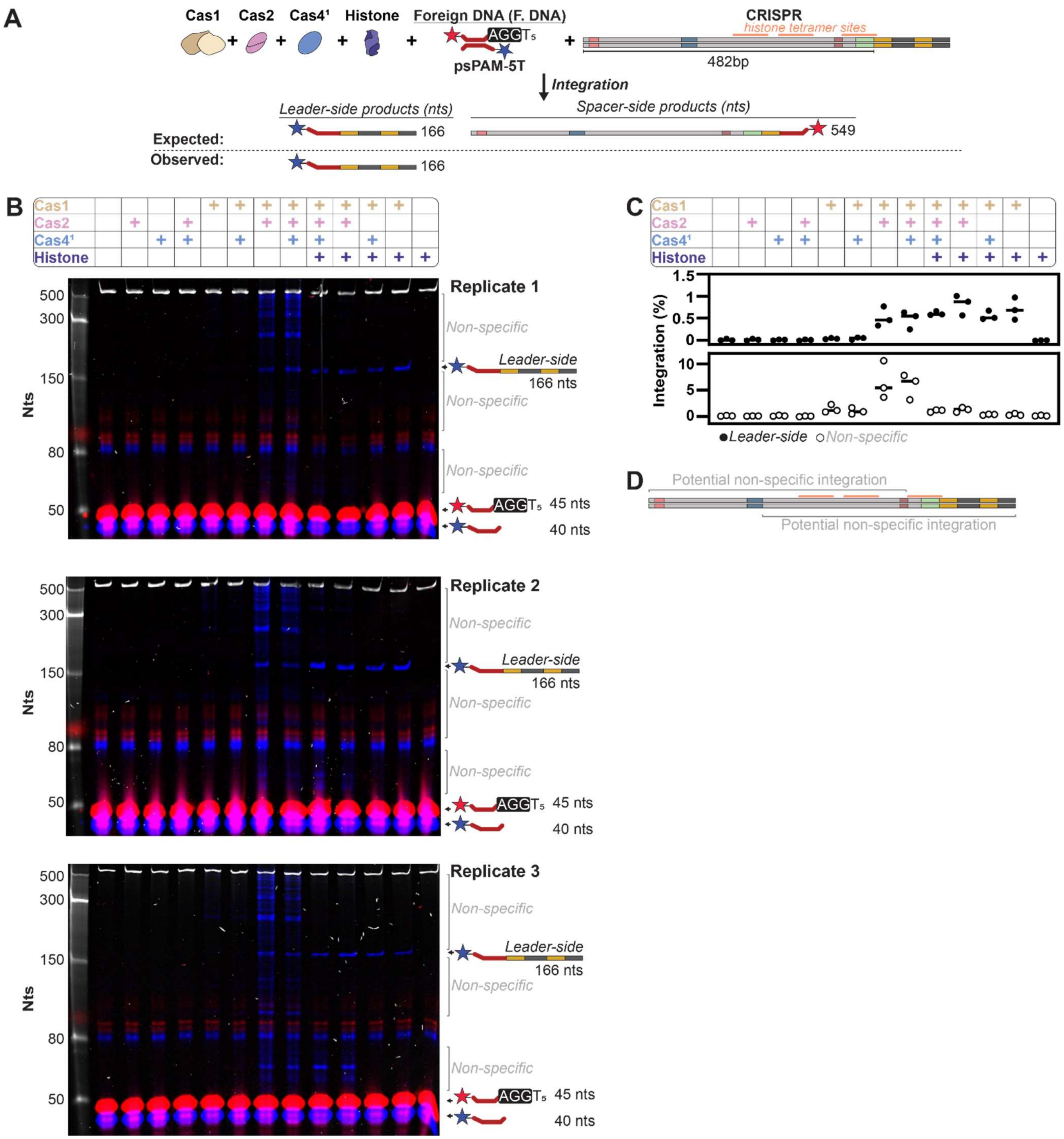

Supplemental Figure S4. *P. furiosus* Histone A promotes Cas1-2-mediated prespacer integration. (**A**) Scheme depicting substrates used to reconstitute CRISPR adaptation in a test tube, as well as the expected and observed integration products. (**B**) Three sets of independently assembled integration reactions resolved on denaturing polyacrylamide gels. (**C**) Quantification of leader- (black circles) or spacer-side (white circles) integration events from all three replicate gels. Individual dots for each triplicate reaction, and their mean, are shown. (**D**) The regions in which non-specific integration events that would give rise to fluorescently labelled products of roughly 250-550 bps in size are shown for each strand of the CRISPR-containing DNA substrate.

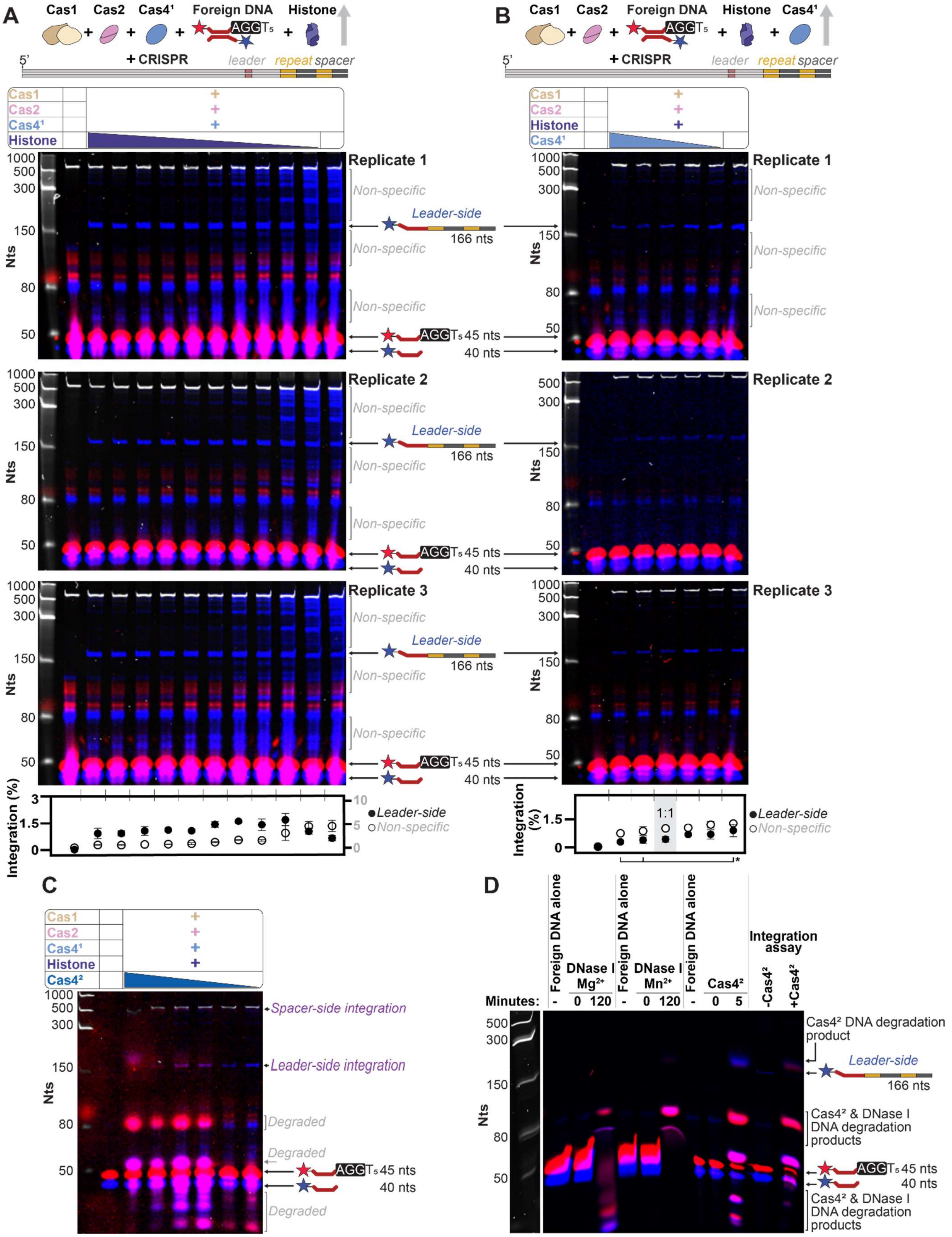

Supplemental Figure S5. Optimization of *in vitro* integration reactions and characterization of cleavage products. (**A**) Scheme of substrates in integration assays to identify an optimal Histone A concentration (top). Integration assays in triplicate (middle). Quantification of leader-side and non-specific integration events

(bottom). Histone A was titrated linearly from 0.5-5 µM. (**B**) Scheme of substrates in integration assays to identify an optimal Cas4[1] concentration (top). Integration assays in triplicate (middle). Quantification of leader-side and non-specific integration events (bottom). Cas4[1] was titrated in the following ratios relative to two Cas1 dimers: 0.25:1, 0.5:1, 1:1, 2:1, and 4:1; Such that the highest concentration of Cas4[1] used was 500 nM. The quantification of reactions containing a 1:1 ratio of one Cas4[1]:two Cas1 dimers is highlighted in grey. Statistical significance was calculated using Dunnett's multiple comparisons test, $*P < 0.05$. (**C**) Integration assays to identify an optimal Cas4[2] concentration. Cas4[2] was titrated in two-fold increments from 2.5-125 nM. (**D**) Prespacer incubated with DNase I in either an Mg^{2+}- or an Mn^{2+}-containing buffer, yields fluorescent products that migrate slower (Mg^{2+}- and Mn^{2+}-facilitated cleavage), or faster (Mg^{2+}-facilitated cleavage). DNase I is known to have different cleavage preferences in the presence of either Mg^{2+} or Mn^{2+} cofactors, and produces one or two nucleotide overhangs in the presence of Mn^{2+} [3]. DNase I is a nonspecific endonuclease that cleaves DNA to release di-, tri- and oligonucleotide products [4,5]. The HEPES buffer used in DNase I assays causes a broadening of the prespacer bands. The last two lanes labeled "Integration Assay" contain Cas1, Cas2, Histone A and CRISPR fragment DNA.

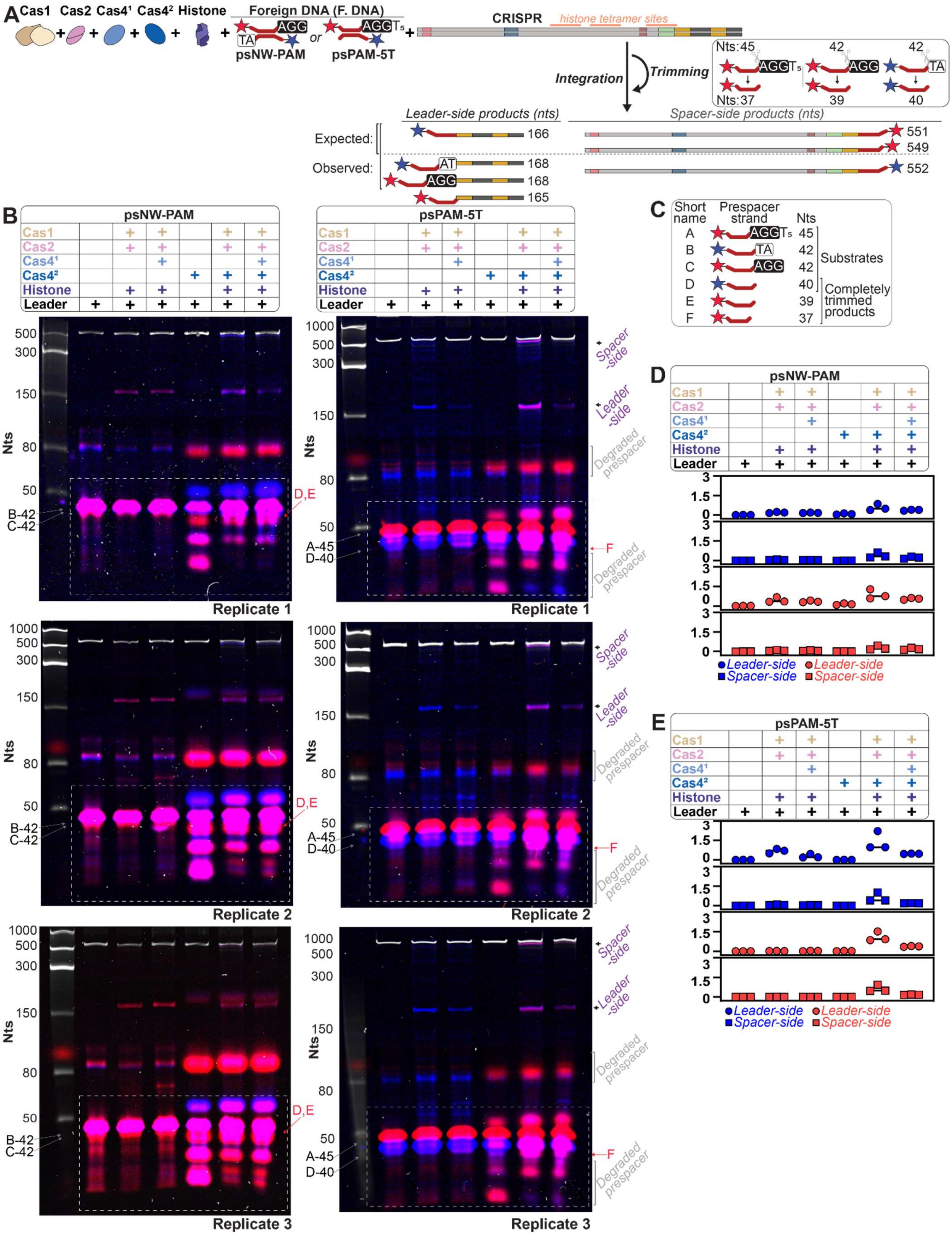

Supplemental Figure S6. Cas4^2 trims prespacers to license spacer-side integration. (**A**) Scheme depicting substrates used to reconstitute CRISPR adaptation in a test tube, as well as the expected and observed integration products. The manner in which the three unique prespacer strands that make up psNW-PAM and

psPAM-5T are hypothesized to be trimmed are shown. (**B**) Three sets of independently assembled integration reactions resolved on denaturing polyacrylamide gels. Reactions in which psNW-PAM (left), and psPAM-5T (right) were used as substrates are shown. A white dashed rectangle is shown for each gel to outline the region used for trimming quantification in Supplemental Figure 7. (**C**) Key for substrate labels used in panel B. (**D**) Quantification of integration events from all three replicate gels in which psNW-PAM was used as a substrate. Leader- (circles) and spacer-side (squares) integration events have been subdivided into those corresponding to integration of the non-PAM (blue) and PAM-containing (red) strands. Individual dots for each triplicate reaction, and their mean, are shown. (**E**) Quantification of integration events from all three replicate gels in which psPAM-5T was used as a substrate. Leader- (circles) and spacer-side (squares) integration events have been subdivided into those corresponding to integration of the non-PAM (blue) and PAM-containing (red) strands. Individual dots for each triplicate reaction, and their mean, are shown.

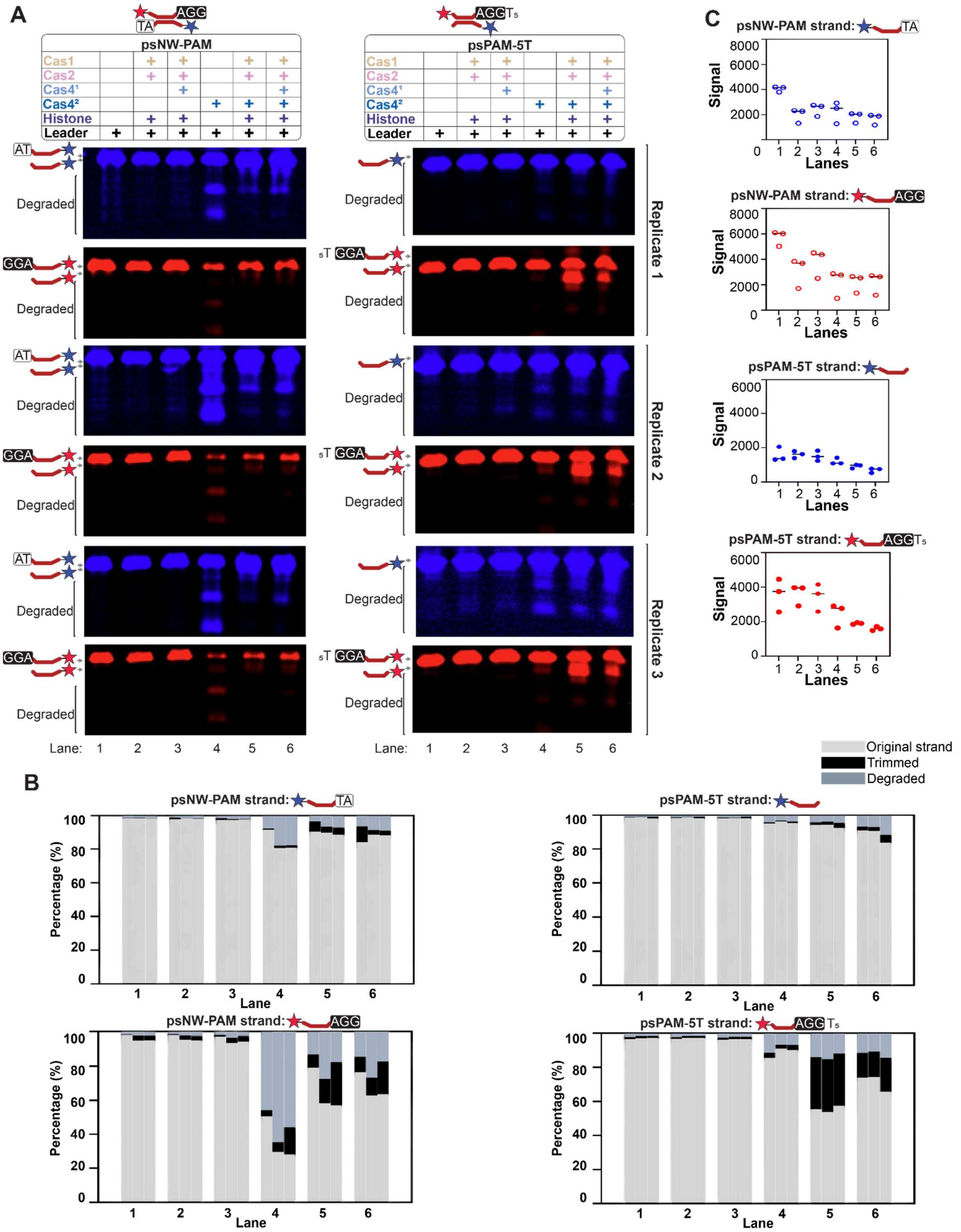

Supplemental Figure S7. Cas4^2 trims each strand of the prespacer to a Cas1-2-protected size. (**A**) Zoomed-in views of the triplicate integration reactions resolved in Supplemental Figure 6. The section of the gels that were boxed in white dashed rectangles, containing the prespacer strands, trimmed and degraded

DNAs are shown. Separate images are shown for the 700 nm (blue) and 800 nm (red) channels to assist in visualization of the trimming of each prespacer strand. (**B**) Quantification of full-length, trimmed and extensively degraded prespacer strands, for each replicate, for both prespacer strands in a duplex are shown. (**C**) Quantification of total signal in the full lane (see Supplemental Figure 4 gel images) for the 700 nm (blue) and 800 nm (red) channels. This quantification shows total prespacer signal tends to decrease slightly in lanes containing Cas4^2 (lanes 4,5, and 6).

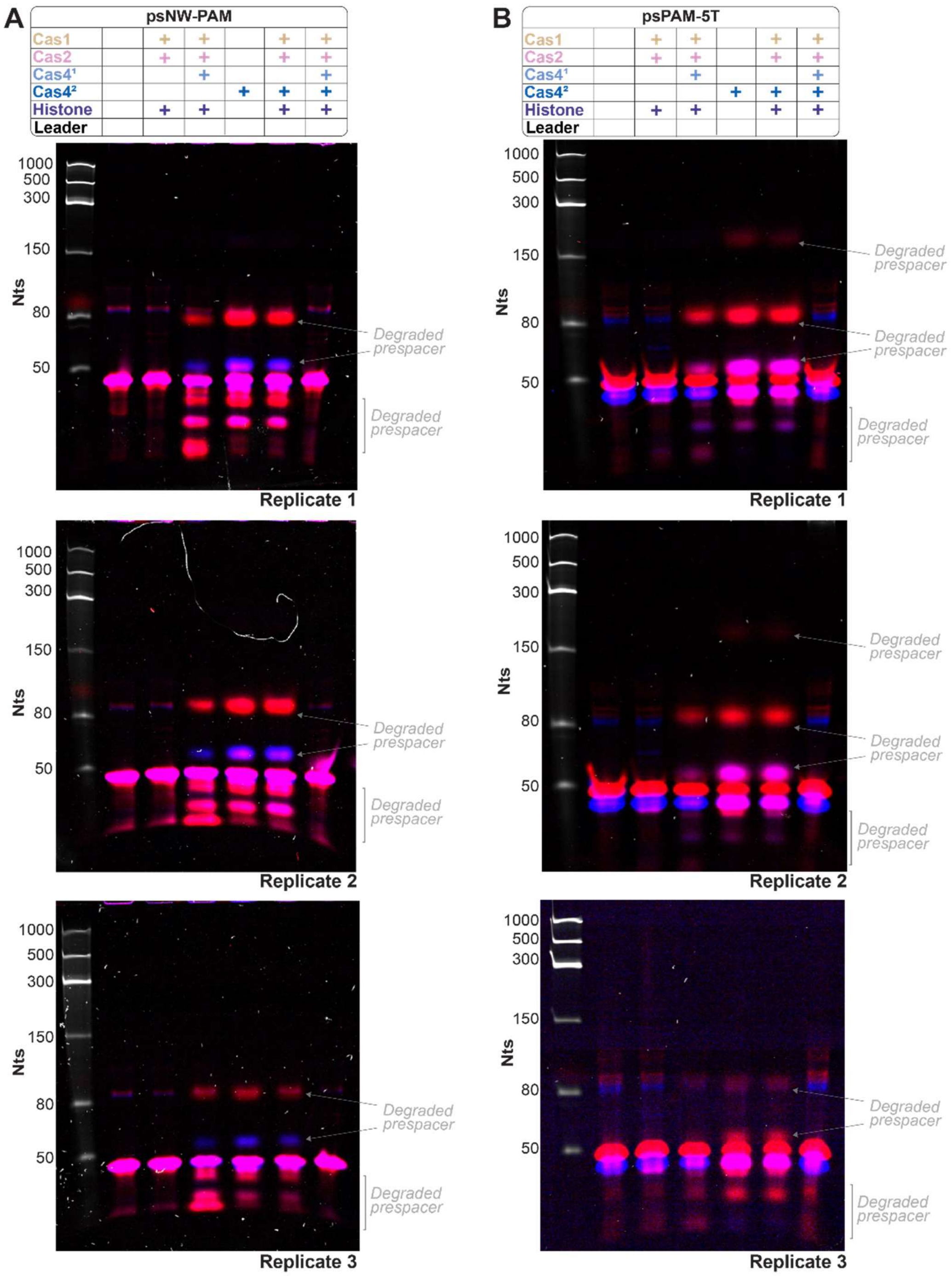

Supplemental Figure S8. Cas4^2 cuts and trims prespacers into fragments that migrate either faster or slower than full length prespacer strands. Three sets of independently assembled, control, integration reactions that lacked CRISPR leader and locus DNA substrate, resolved on denaturing polyacrylamide gels. Reactions in which (**A**) psNW-PAM and (**B**) psPAM-5T were used as substrates are shown. No CRISPR substrate was included, therefore no integration can occur. Slow migrating bands that appear to migrate at 60 bps, 90 bps and 200 bps are products of Cas4^2-mediated cleavage of the prespacer strand. Also see Supplemental Figure S5 for an example of an unrelated DNase (DNase I) producing slower migrating cleavage products.

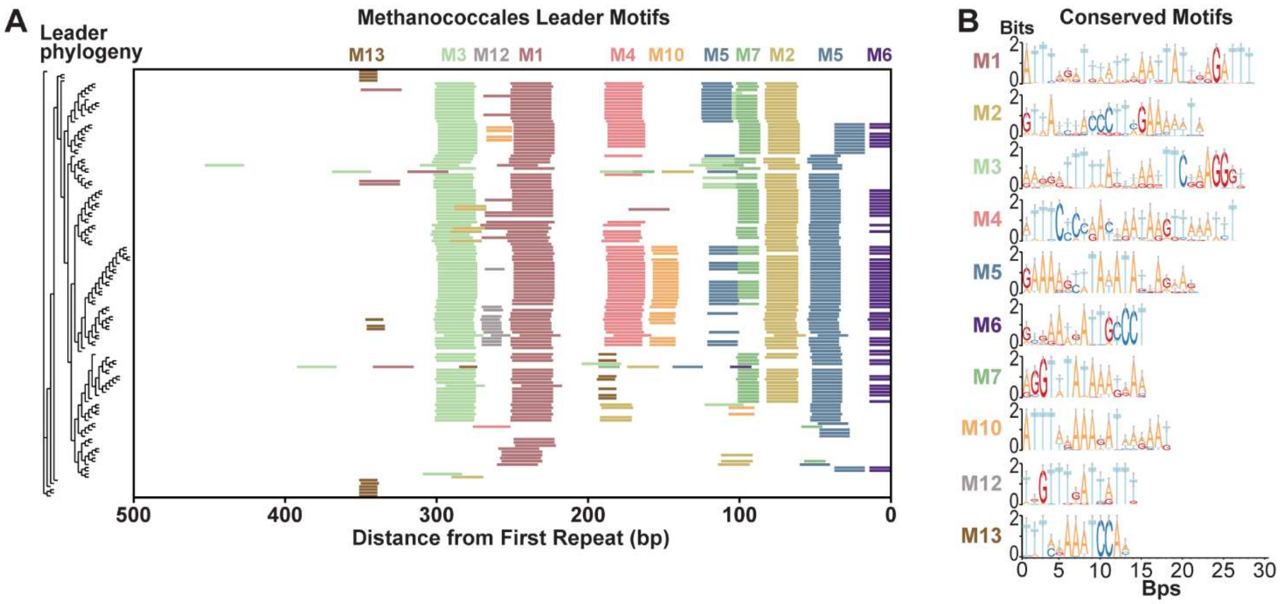

Supplemental Figure S9. *Methanococcales* leaders contain conserved motifs up to 350 bps upstream of the CRISPR. (**A**) A phylogenetic tree generated from the alignment of 500 bps of CRISPR leader and the first repeat from 193 non-redundant sequences. The CRISPR leader corresponding to each terminal node of the tree, is represented in the plot as a horizontal line (not shown) extending from the node, and significant conserved motif occurrences are plotted as colored bars. Different motifs are depicted by unique colors and numbered from most significant (smaller number) to least (larger number). Motif colors and numbers are shown above the plot. (**B**) Sequence logos of each motif.

A

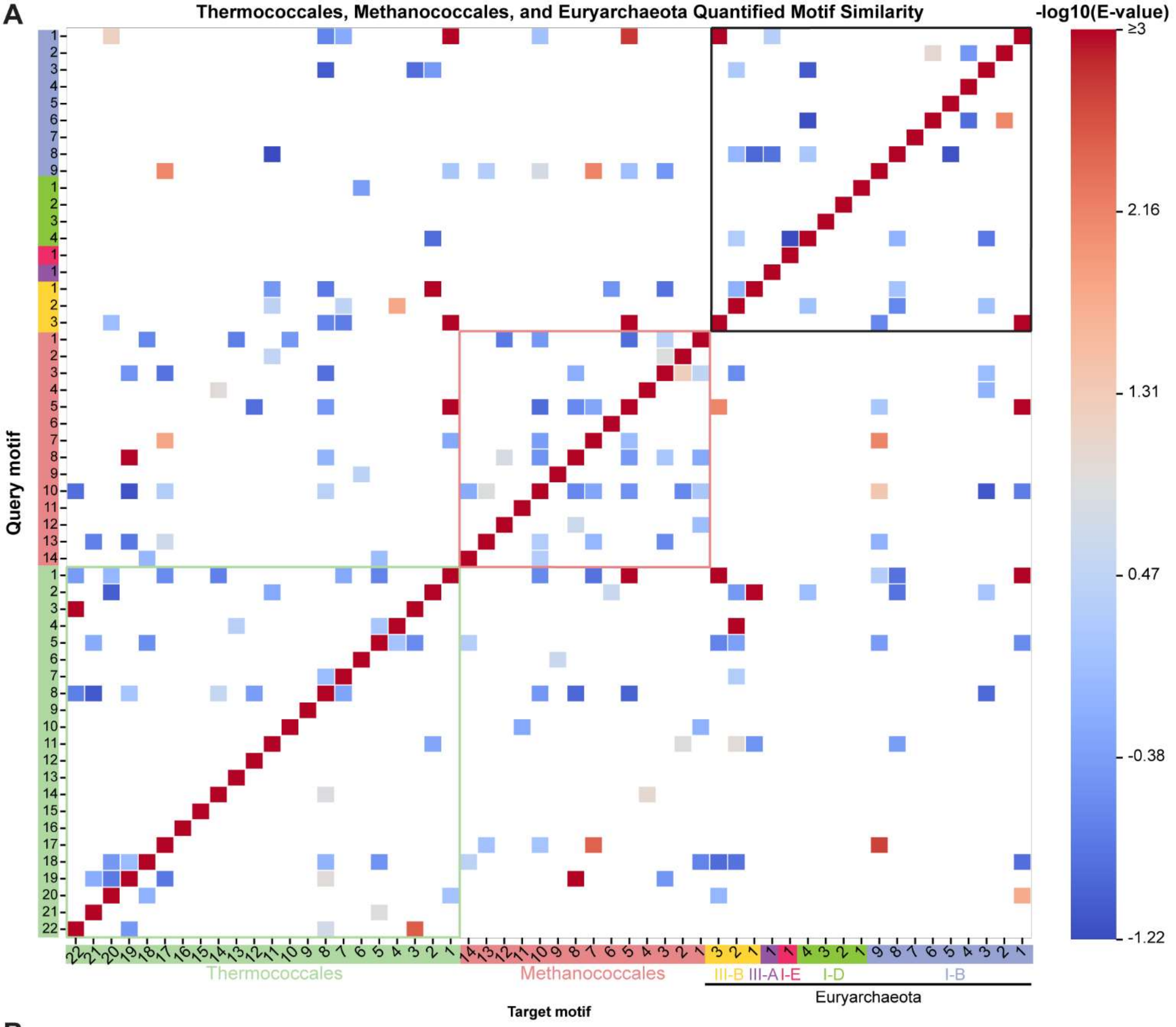

B

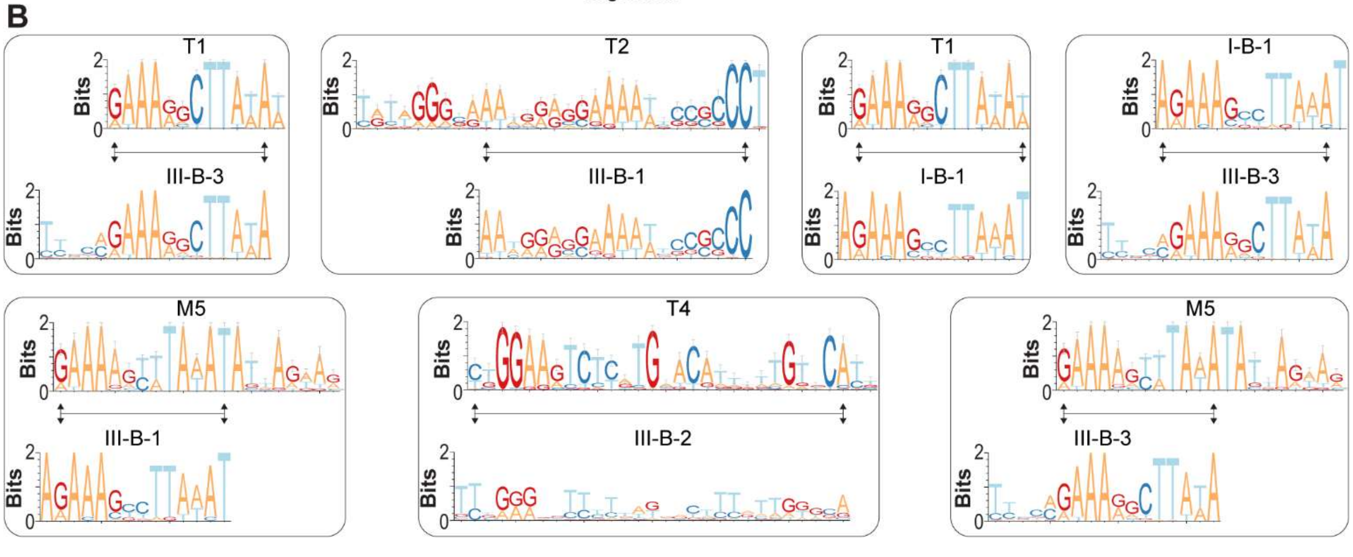

Supplemental Figure S10. Tomtom analysis reveals some leader motifs are conserved between the archaeal orders *Thermococcales* and *Methanococcales*. (**A**) An all-to-all comparison of the motifs identified in *Thermococccales* and *Methanococcales* in this paper, with CRISPR leader motifs previously identified across Euryarchaeota [6]. Motif similarity was calculated using Tomtom [7], and a threshold of -log(E-value) ≥ 3 was

chosen to identify significant motif matches (**Supplemental Data S5**). (**B**) Stacked motif logos are shown to visualize significant matches between *Thermococccales* and *Methanococcales* motifs, and to previously reported motifs [6]. Motifs identified in this paper have error bars shown.

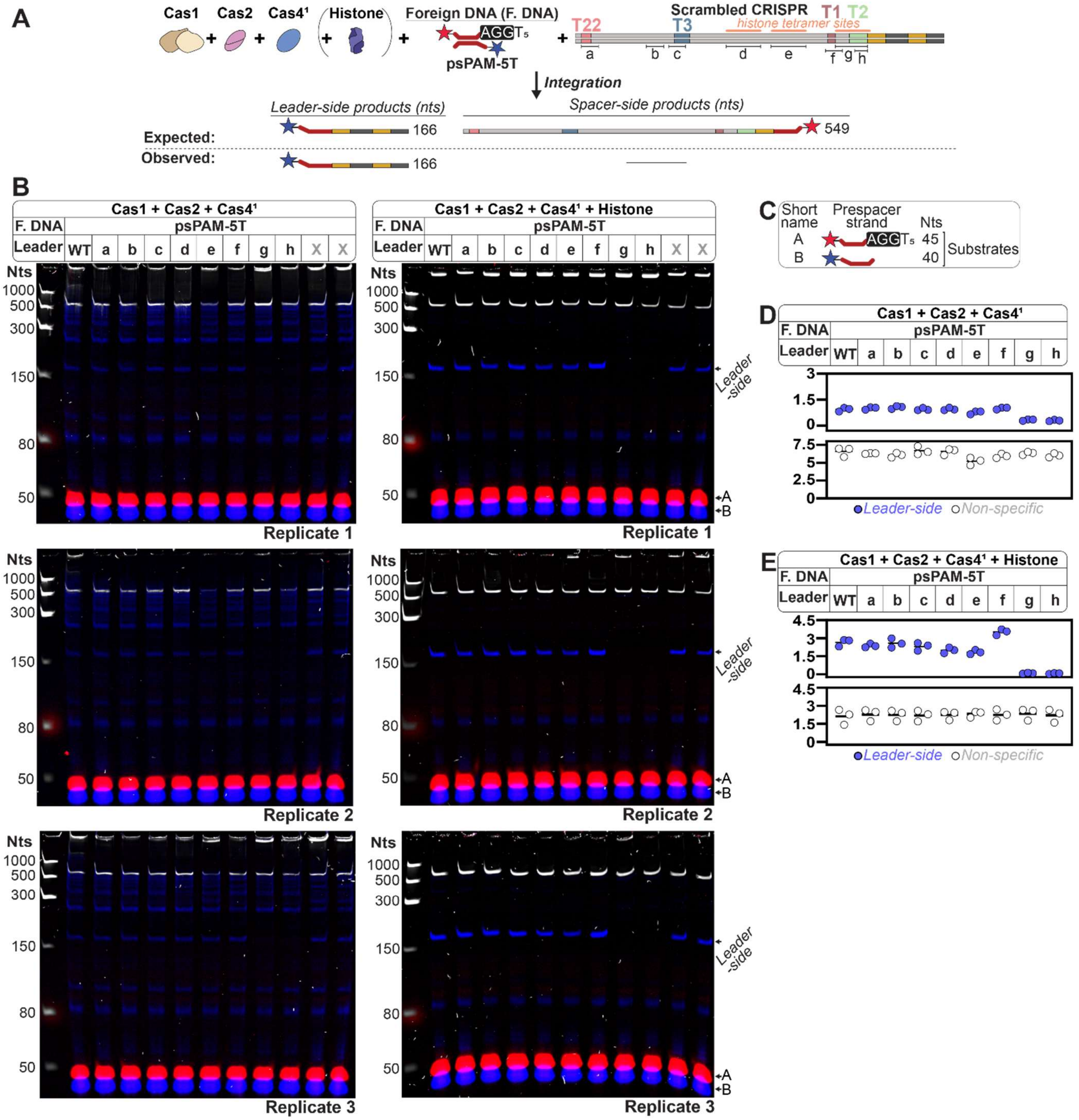

Supplemental Figure S11. Leader motifs facilitate histone-mediated and histone-independent integration at the first repeat. (**A**) Scheme of integration reactions conducted either with or without Histone A (bracketed), with the psPAM-5T prespacer, into CRISPR fragments with either wildtype leader, or a leader containing scrambled DNA sequence motifs. All expected and observed integration products are depicted. A stock of Cas4[1] that was slightly less pure, but which appeared to contain a higher concentration of iron was used for these assays and the associated Figure 3. (**B**) Three sets of independently assembled integration reactions resolved on denaturing polyacrylamide gels. Reactions conducted in the absence (left) or presence (right) of Histone A are shown. The X labels signify two lanes that were not further analyzed for this manuscript. (**C**) Key for substrate labels used in panel B. (**D**) Quantification of integration events from all three replicate

reactions conducted in the absence of Histone A. Leader-side integration (blue circles) and non-specific integration (white circles) of the non-PAM strand are shown. Spacer-side integration events could not be confidently assigned in these reactions. Individual dots for each triplicate reaction, and their mean, are shown. (**E**) Quantification of integration events from all three replicate reactions conducted in the presence of Histone A. Leader-side integration (blue circles) and non-specific integration (white circles) of the non-PAM strand are shown. Spacer-side integration events could not be confidently assigned in these reactions. Individual dots for each triplicate reaction, and their mean, are shown.

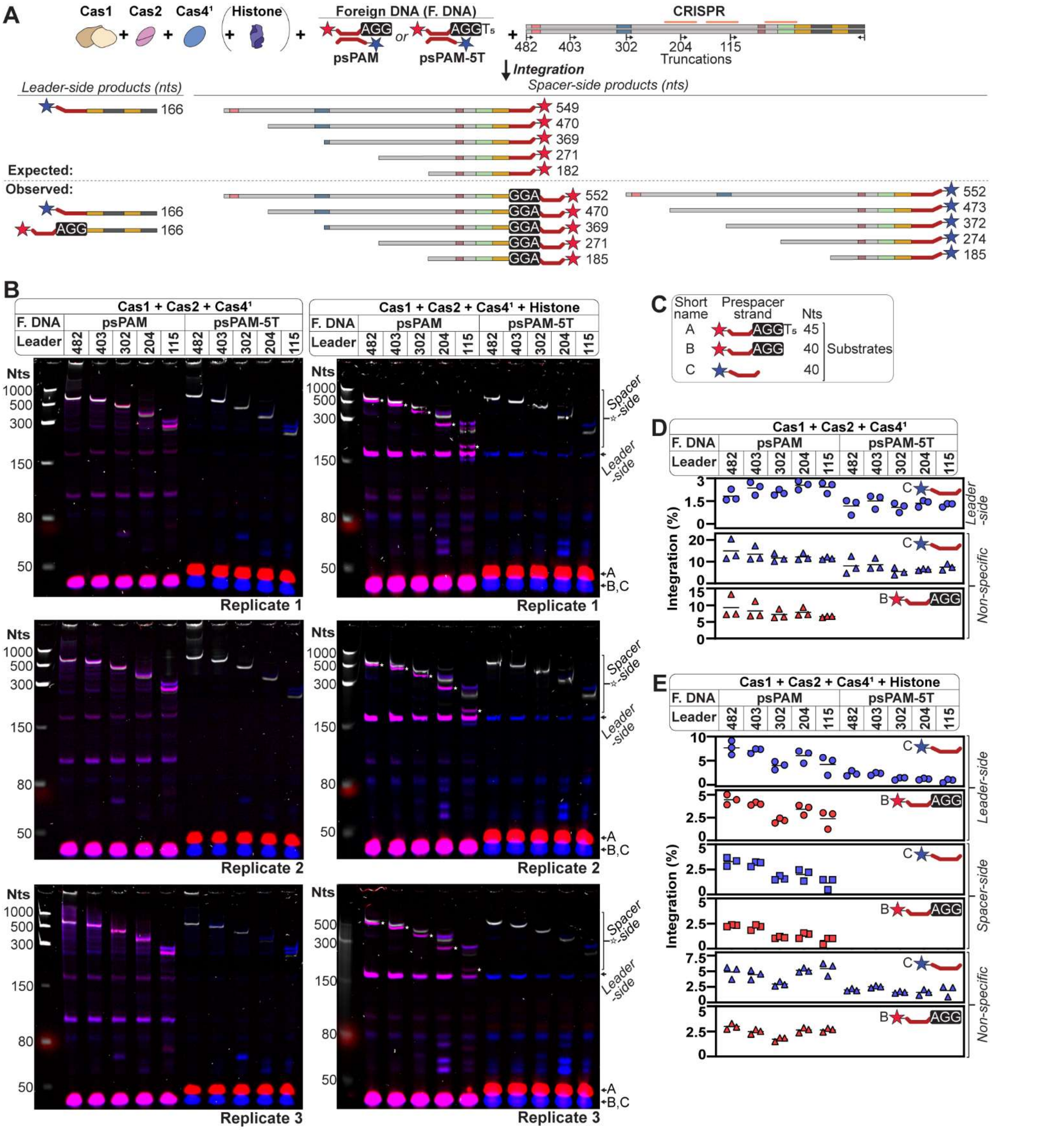

Supplemental Figure S12. Histones and up to 403 bps of leader promote full-site integration of large prespacers by Cas1-2. (**A**) Scheme of integration reactions conducted either with or without Histone A (bracketed), with either the psPAM or psPAM-5T prespacers, into CRISPR fragments containing either full-length or truncated leaders. All expected and observed integration products are depicted. (**B**) Three sets of independently assembled integration reactions resolved on denaturing polyacrylamide gels. Reactions conducted in the absence (left) or presence (right) of Histone A are shown. (**C**) Key for substrate labels used in panel B. (**D**) Quantification of integration events from all three replicate reactions conducted in the absence of Histone A. Leader-side (circles) and non-specific (triangles) integration events have been subdivided into those

corresponding to integration of the non-PAM (blue) and PAM-containing (red) strands. Spacer-side integration events could not be confidently assigned in these reactions. Individual dots for each triplicate reaction, and their mean, are shown. (**E**) Quantification of integration events from all three replicate reactions conducted in the presence of Histone A. Leader- (circles), spacer-side (squares), and non-specific (triangles) integration events have been subdivided into those corresponding to integration of the non-PAM (blue) and PAM-containing (red) strands. Spacer-side integration products could only be confidently assigned to the psPAM substrate integrated in the presence of histones, and therefore was only quantified and plotted for such reactions. Individual dots for each triplicate reaction, and their mean, are shown.

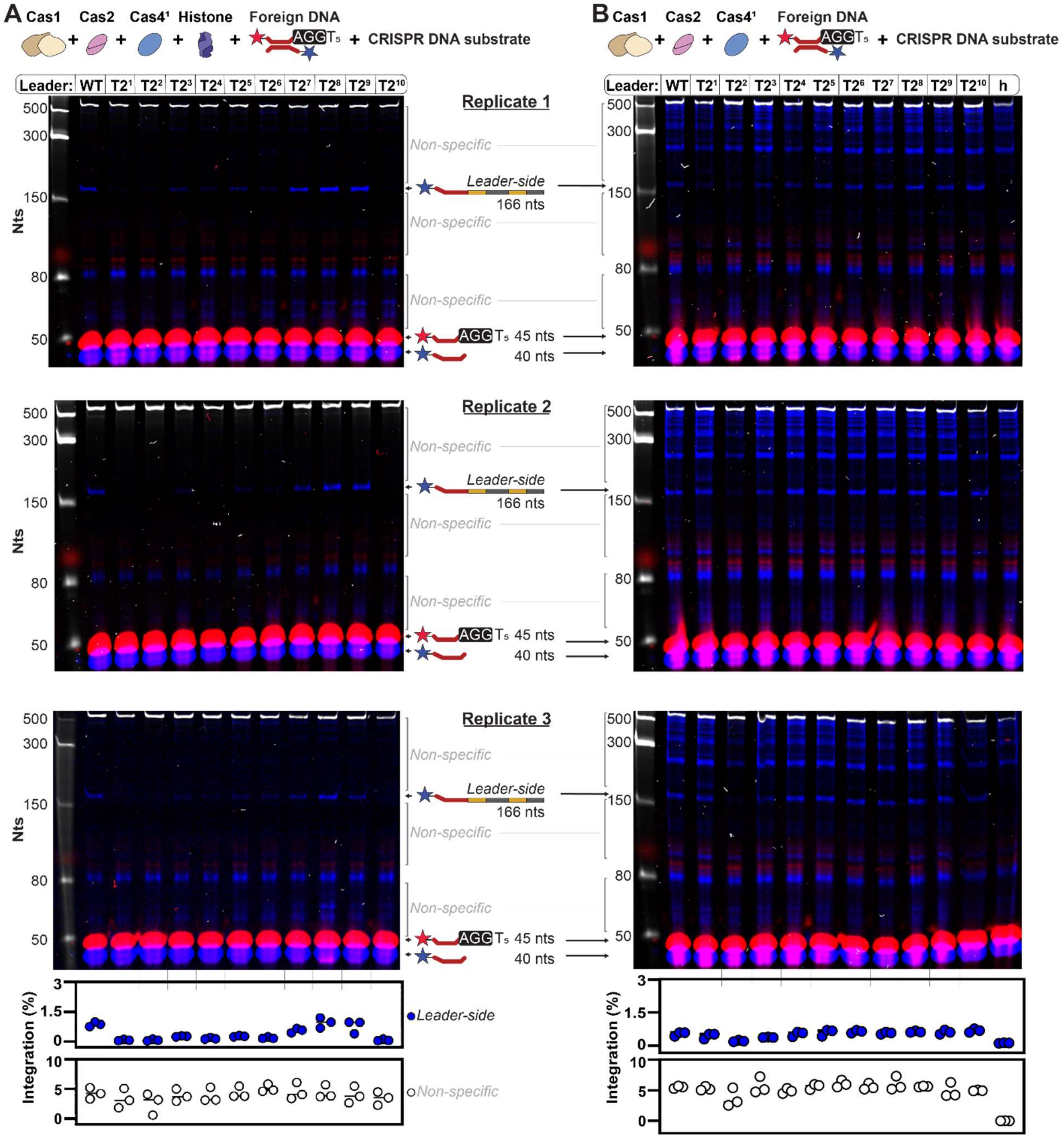

Supplemental Figure S13. Tiled mutations across the T2 motif reveal positions required for histone-dependent and histone-independent integration. Three sets of independently assembled integration reactions resolved on denaturing polyacrylamide gels. Reactions conducted in the absence (left) or presence (right) of Histone A are shown.

REFERENCES

1. Zhang, J., Kasciukovic, T. & White, M. F. The CRISPR Associated Protein Cas4 Is a 5' to 3' DNA Exonuclease with an Iron-Sulfur Cluster. *PLoS ONE* **7**, e47232 (2012).
2. Watts, E. A. *et al.* Histones direct site-specific CRISPR spacer acquisition in model archaeon. *Nat. Microbiol.* **8**, 1682–1694 (2023).
3. Green, M. R. & Sambrook, J. *Molecular Cloning: A Laboratory Manual*. (Cold Spring Harbor laboratory press, Cold Spring Harbor, 2012).
4. Kunitz, M. CRYSTALLINE DESOXYRIBONUCLEASE. *J. Gen. Physiol.* **33**, 349–362 (1950).
5. Vanecko, S. & Laskowski, M. Studies of the Specificity of Deoxyribonuclease I. *J. Biol. Chem.* **236**, 3312–3316 (1961).
6. Buyukyoruk, M., Krishna, P., Santiago-Frangos, A. & Wiedenheft, B. Discovery of Diverse CRISPR Leader Motifs, Putative Functions, and Applications for Enhanced CRISPR Detection and Subtype Annotation. *CRISPR J.* crispr.2024.0093 (2025) doi:10.1089/crispr.2024.0093.
7. Gupta, S., Stamatoyannopoulos, J. A., Bailey, T. L. & Noble, W. S. Quantifying similarity between motifs. *Genome Biol.* **8**, R24 (2007).
