# Supplementary material for: Archaeal histones coordinate conserved DNA sequence motifs to promote CRISPR adaptive immunity
Source: Nucleic Acids Res. 2026 Nov 10;54(21):gkag1172. doi: 10.1093/nar/gkag1172 (PMC13748974; doi:10.1093/nar/gkag1172)
Supplement: Supplementary file 1 [file gkag1172_supplemental_files.zip › Supplemental_Table_S1.docx]

**Supplemental Table S1. Oligonucleotides used in this manuscript.**

| Oligo Name | Sequence (5’ to 3’) |
| --- | --- |
| psPAM (sense) | /[5IRD700](https://www.idtdna.com/site/Catalog/Modifications/Product/1723)/AACTTATATAAGCAATACGGAACTTATGAGAACTATTAGG |
| psPAM (anti-sense) | /[5IRD800](https://www.idtdna.com/site/Catalog/Modifications/Product/1724)/CCTTTATCTTCTCATAAGTTCCGTATTGCTTATATTTCAA |
| psNW-PAM (sense) | /[5IRD700](https://www.idtdna.com/site/Catalog/Modifications/Product/1723)/ATAACTTATATAAGCAATACGGAACTTATGAGAACTATTAGG |
| psNW-PAM (anti-sense) | /[5IRD800](https://www.idtdna.com/site/Catalog/Modifications/Product/1724)/CCTTTATCTTCTCATAAGTTCCGTATTGCTTATATTTCAAAT |
| psPAM-5T (sense) | /[5IRD700](https://www.idtdna.com/site/Catalog/Modifications/Product/1723)/ATAACTTATATAAGCAATACGGAACTTATGAGAACTATTAGGTTTTT |
| psPAM-5T (anti-sense) | /[5IRD800](https://www.idtdna.com/site/Catalog/Modifications/Product/1724)/CCTTTATCTTCTCATAAGTTCCGTATTGCTTATATTTCAA |
| Primers | |
| PfuCR7_rev2 | CTAATCGAAGACTTCGTAAGAGATAACGA |
| PfuCR7_520L_F | TCTCTCAACTCCCACATCGTA |
| PfuCR7_482L_F | TGTGAAACACCCAAAAATTTTTCGATAC |
| PfuCR7_403F2-asf | GAAAATAAAGGTTTTTGAGGGATTAGGG |
| PfuCR7_302F-asf | CGAAAACTATCGAATAATATTTTAAGAGG |
| PfuCR7_204F2-asf | CTAGTTAAGTCTTCTCCTGGGATAGG |
| PfuCR7_115L_F | GGGAGTTGGTTTGTTTATAAATGGG |
| PfuCR7_Scr_482F_L | TGTGAAACACAACCTTCTATATTTATTCAAAAAAGC |
| T3_WT_15bpflank_F | GCCCCGTAAGGCTTATAAATTCGAGTGATGTAGTTACTCCGTAGGAGGATTGGGGC |
| T3_WT_15bpflank_R | TCTTTTGTAGTCTTATTGGAACAGGGGGCTTTTTTGCCCCAATCCTCCTACG |
| T3_WT_M2_Scr_15bpflank_F | GCCCCGTAAGGCTTATAAATTCGAGTGATGTAGTTACTCCGTAGGAGCCG |
| T3_WT_M2_Scr_15bpflank_R | TCTTTTGTAGTCTTATTGGAACAATCTACTCCTTGTGTCGGCGGCTCCTACGGAGTAAC |
