# Supplementary material for: Archaeal histones coordinate conserved DNA sequence motifs to promote CRISPR adaptive immunity
Source: Nucleic Acids Res. 2026 Nov 10;54(21):gkag1172. doi: 10.1093/nar/gkag1172 (PMC13748974; doi:10.1093/nar/gkag1172)
Supplement: Supplementary file 1 [file gkag1172_supplemental_files.zip › Supplemental_Table_S2.docx]

**Supplemental Table S2. Plasmids used in this manuscript.**

| Plasmid | Description | Antibiotic selection | Addgene Number |
| --- | --- | --- | --- |
| WT_PfuCR7-2R-2S_pUC57 | Encodes 520bp of leader, 2 repeats, and 2 spacers of CRISPR 7 array from P. furiosus system | Ampicillin | 244112 |
| 472-443Scr_PfuCR7-2R-2S_pUC57_meme4_1 | Scrambled a region found 472-443 basepairs upstream of the 1st repeat to test its role in facilitating integration. | Ampicillin | 244113 |
| 329-300Scr_PfuCR7-2R-2S_pUC57_meme4_2 | Scrambled a region found 329-300 basepairs upstream of the 1st repeat to test its role in facilitating integration. | Ampicillin | 244114 |
| 234-176Scr_PfuCR7-2R-2S_pUC57_T1 | Scrambled a region found 234-176 basepairs upstream of the 1st repeat to test its role in facilitating integration. | Ampicillin | 244115 |
| 160-101Scr_PfuCR7-2R-2S_pUC57_T2 | Scrambled a region found 160-101 basepairs upstream of the 1st repeat to test its role in facilitating integration. | Ampicillin | 244116 |
| 54-1Scr_PfuCR7-2R-2S_pUC57_T3 | Scrambled a region found 54-1 basepairs upstream of the 1st repeat to test its role in facilitating integration. | Ampicillin | 244117 |
| 366-337Scr_PfuCR7-2R-2S_pUC57_meme8 | Scrambled a region found 366-337 basepairs upstream of the 1st repeat to test its role in facilitating integration. | Ampicillin | 244118 |
| 70-41Scr_PfuCR7-2R-2S_pUC57_meme10 | Scrambled a region found 70-41 basepairs upstream of the 1st repeat to test its role in facilitating integration. | Ampicillin | 244119 |
| 22-1Scr_PfuCR7-2R-2S_pUC57_meme2 | Scrambled a region found 22-1 basepairs upstream of the 1st repeat to test its role in facilitating integration. | Ampicillin | 244120 |
| SUMOtagged_PfuCas1_pRSFDuet-1 | Encodes for an N-term translational ramp with a GSS linker with a 10x His tag linked by GSG followed by SUMO tagged Pfu type I-B Cas1. | Kanamycin | 244121 |
| PfuHistA_pRSFDuet-1 | Encodes for Histone A from Pfu. | Kanamycin | 244122 |
| PfuCas2(NoTag)_pETDuet-1 | Encodes for the Pfu type I-B Cas2 | Ampicillin | 244123 |
| SUMOtagged_PfuCas4^1^_pET-28a(+) | Encodes for a N-term 6x His Tag linked by SSG to a Fusion of SUMO and the Pfu type I-B Cas4^1^. | Kanamycin | 244124 |
| SUMOtagged_PfuCas4^2^_pET-28a(+) | Encodes for a N-term 6x His Tag linked by SSG to a Fusion of SUMO and the Pfu type I-B Cas4^2^. | Kanamycin | 244125 |
| 1-3tra_PfuCR7 | Transversion mutation of a region found 3-1 basepairs upstream of the 1st repeat to test its role in facilitating integration. | Ampicillin | 260946 |
| 4-7tra_PfuCR7 | Transversion mutation of a region found 7-4 basepairs upstream of the 1st repeat to test its role in facilitating integration. | Ampicillin | 260947 |
| 4-30tra_PfuCR7 | Transversion mutation of a region found 30-4 basepairs upstream of the 1st repeat to test its role in facilitating integration. | Ampicillin | 260948 |
| 8-11tra_PfuCR7 | Transversion mutation of a region found 11-8 basepairs upstream of the 1st repeat to test its role in facilitating integration. | Ampicillin | 260949 |
| 12-15tra_PfuCR7 | Transversion mutation of a region found 15-12 basepairs upstream of the 1st repeat to test its role in facilitating integration. | Ampicillin | 260950 |
| 16-19tra_PfuCR7 | Transversion mutation of a region found 19-16 basepairs upstream of the 1st repeat to test its role in facilitating integration. | Ampicillin | 260951 |
| 20-23tra_PfuCR7 | Transversion mutation of a region found 23-20 basepairs upstream of the 1st repeat to test its role in facilitating integration. | Ampicillin | 260952 |
| 24-27tra_PfuCR7 | Transversion mutation of a region found 27-24 basepairs upstream of the 1st repeat to test its role in facilitating integration. | Ampicillin | 260953 |
| 28-30tra_PfuCR7 | Transversion mutation of a region found 30-28 basepairs upstream of the 1st repeat to test its role in facilitating integration. | Ampicillin | 260954 |
| MidHisTopToProx | Mutation that replaces the proximal histone binding site with the middle histone binding site, to determine whether any histone binding site can be used at the proximal site. | Ampicillin | 260955 |
| SENP2 | SUMO Protease | Kanamycin | 261192 |
